# Mapping global bee research with traits and plant-pollinator interaction networks

**DOI:** 10.1038/s41598-026-41830-7

**Published:** 2026-03-10

**Authors:** Miles Liam Nesbit, Cecilia Montauban, Francis Windram, Miguel Santiago Bailey Pérez, William O. H. Hughes, Dave Goulson, Richard J. Gill, Peter Graystock

**Affiliations:** 1 Department of Life Sciences, Georgina Mace Centre for the Living Planet, Imperial College London, Silwood Park, Ascot, Berkshire, UK; 2 The Grantham Institute, Imperial College London, South Kensington, London, UK; 3 School of Life Sciences, University of Sussex, Brighton, UK

**Keywords:** Bees, Pollinators, Conservation, Policy, Plant–pollinator networks, Trait analysis, Ecology, Ecology, Zoology

## Abstract

Bees sustain key functions in natural ecosystems and agricultural landscapes, yet our understanding of their ecology is typically informed from studies concentrated on a few model taxa. To reveal how this may be biasing our understanding of bee responses and function in the environment we quantify global patterns of research attention across 69,682 bee-related publications to test whether research effort aligns with plant-pollinator network centrality, trait variation, public interest, and socio-economic context. Human managed bees take up most of the research effort; importantly this trend has been increasing over time. Plant–pollinator network centrality is unrelated to research effort; here we reveal genera with high centrality but low research attention as prime candidates for future study. Both pollinator management and sociality have an impact on research effort. Excluding *Apis* and *Bombus* (the most traditionally researched genera), managed bee genera are the focus of twice as many papers as wild genera, with the managed share rising over time. Our study reveals and quantifies persistent global research biases and highlights the need for monitoring, risk assessment, and policies that target neglected yet structurally central genera in plant-pollinator interaction networks.

**Supplementary Information:**

The online version contains supplementary material available at 10.1038/s41598-026-41830-7.

## Introduction

Insect declines threaten ecosystem function and human well-being. Multiple syntheses report widespread reductions in insect abundance and diversity, with cascading effects on food webs, nutrient cycling, and food security^1–4^. Bees (Hymenoptera: Anthophila), for instance, provide a large share of animal-mediated pollination in both wild and agricultural plant communities across the world^5,6^. Yet, despite there being over 20,000 species of bees worldwide, research has largely concentrated on only a small fraction of them. For example, the managed European honey bee (*Apis mellifera*), the buff-tailed bumblebee (*Bombus terrestris*), and the common eastern bumblebee (*Bombus impatiens*) have received significant attention due to their commercial value and cultural prominence^7–11^. Bias audits and bibliometric syntheses show that effort clusters around genera such as *Apis* and *Bombus* across Europe and North America, and that topics such as pollination services, ecotoxicology, and disease feature prominently^2,12–18^.

Agenda-setting forces like public perception and economic interests are partly responsible for narrowing scientific focus and conservation policy towards a few taxa. Research funding is often focused on charismatic or commercially valuable species, most notably honey bees, where concerns about high rates of colony mortality continue to drive intensive research and regulatory action on neonicotinoids^19–22^. This ‘charisma bias’ further elevates highly visible species, such as bumblebees, concentrating attention on a small set of taxa and shaping what is measured, published, and ultimately what threats are regulated^23,24^. Such a narrow focus is problematic because it risks skewing inference about ecosystem functioning and vulnerability. Managed generalist social bees, like *Apis* and *Bombus*, often dominate pollination studies because they are abundant, easily managed and accessible for research, but many vital interactions and ecosystem processes are maintained by less-studied wild bees^25–28^. Solitary bees, including many ground-nesting taxa, illustrate these blind spots clearly: they face different stressors, life histories, and play unique ecological roles, yet they remain underrepresented in research on pesticide risk, disease dynamics, and habitat change^29,30^. Unless research effort covers the full spectrum of ecological roles of diverse bee groups, conservation, policy, and agricultural management will continue to overlook many species that are critical to sustaining pollination systems^27,31^.

The relationships between bees and flowering plants form the backbone of healthy ecosystems and productive agriculture. These relationships can be mapped using interaction networks to understand which bee species are most important for pollination in plant communities^32–35^. These bipartite (two-level) networks provide a means to summarise bee-plant links and quantify a taxon’s structural position within described networks^32,33,36^. Importantly, these networks and their associated metrics often provide a quantification of visitation between bees and plants, rather than direct measures of pollination effectiveness^32,34^. This distinction is necessary because a record of visitation does not confirm pollen transfer, and network metrics are influenced by both abundance and sampling^33,35^. Nonetheless, confirming these ecological links provides the necessary baseline for further research, and helps identify which bees are generalists (visiting, and potentially pollinating, a wide variety of plants), and which are unique specialists, providing potential pollination services to unique plants that few others visit. With plant-pollinator interaction networks (hereafter ‘interaction networks’) we can also examine the importance of different pollinators. For instance, we can assess which potential pollinators have the largest ‘strength’ in the system, i.e., which pollinators are linked to the most flower species, that might therefore rely on them for pollination^25,26,28,31^. Pollinator species with high strength (high centrality in the network, i.e., many interactions with a variety of plants) can be vital for plant systems in any ecosystem (wild or managed/agricultural). These central pollinators are the most efficient ‘units of redundancy’ in a plant-pollinator network; by interacting with a large variety of plants, they create the necessary overlap to ensure that pollination services continue even as other species populations shift or decline.

Network structure is not static: extinctions, climate change, and land-use change can cut interactions, shift partners, and weaken redundancy, with documented consequences for pollination function through time^37–41^. Network centrality (species strength) can therefore serve as a proxy for the structural importance of pollinators within interaction networks, despite the important caveats mentioned, and the reality that communities are shifting under changing environmental conditions.

To understand why certain bees play these roles, we can examine their functional traits e.g., the physical and life history characteristics that define their ecosystem function and vulnerability^42–45^. For example, a bee’s body size dictates its foraging range and dispersal ability, influencing how effectively it can visit fragmented plant populations^30,46^. Similarly, its tongue length determines which flowers it can access, shaping its niche within the community^26,28^. Life history traits, such as whether a bee is social or solitary, where it nests (e.g., in the ground or in cavities), and whether it is a managed species, also offer insights into its vulnerability and contribution to pollination^7,29,30,47–49^.

Given the large extent of bee research conducted, syntheses to date have focused on how research attention maps onto taxonomy (i.e., which species have been most studied) and have confirmed persistent geographic skew toward Europe and North America^12,13,17^. A key remaining knowledge gap is how this research attention maps onto ecosystem functionality and the potential roles that bees play in natural and anthropogenic landscapes. We still lack a global quantitative assessment of how bee research is geographically and economically structured, what aspects of trait space are being overlooked, and whether our focus adequately covers the diversity of important pollination roles across ecological networks.

Here, we quantify global patterns of research attention across 69,682 bee-related publications (1975–2023), using machine-learning–derived publication counts that were independently scored and validated by manual assessment. Accounting for the pronounced temporal increase in publication volume and standardising comparisons across networks and trait dimensions, we test whether research effort aligns with species’ network centrality, functional trait space, public interest, and socio-economic context. We evaluate whether scientific attention tracks ecological centrality by relating genus-level research to interaction-network metrics and assess how research effort covers the ecological trait space of bee communities. We analyse the distribution of research across taxa to determine if effort is disproportionately concentrated on specific genera, such as *Apis* and *Bombus*, relative to biodiversity and public popularity. We investigate how management and life history traits structure research priorities for the broader bee community. Finally, we test whether scientific attention tracks ecological centrality by relating genus-level attention to plant-pollinator interaction-network metrics of contribution and uniqueness, testing robustness across different datasets and metrics. Together, these analyses identify where attention is likely to be misaligned with ecological leverage and provide a roadmap to focus research and policy on the taxa and geographic regions that most sustain pollination, biodiversity, and food security.

## Results

### Research effort is decoupled from plant-pollinator visitation network centrality and covers a small portion of overall trait space

**Fig. 1:**
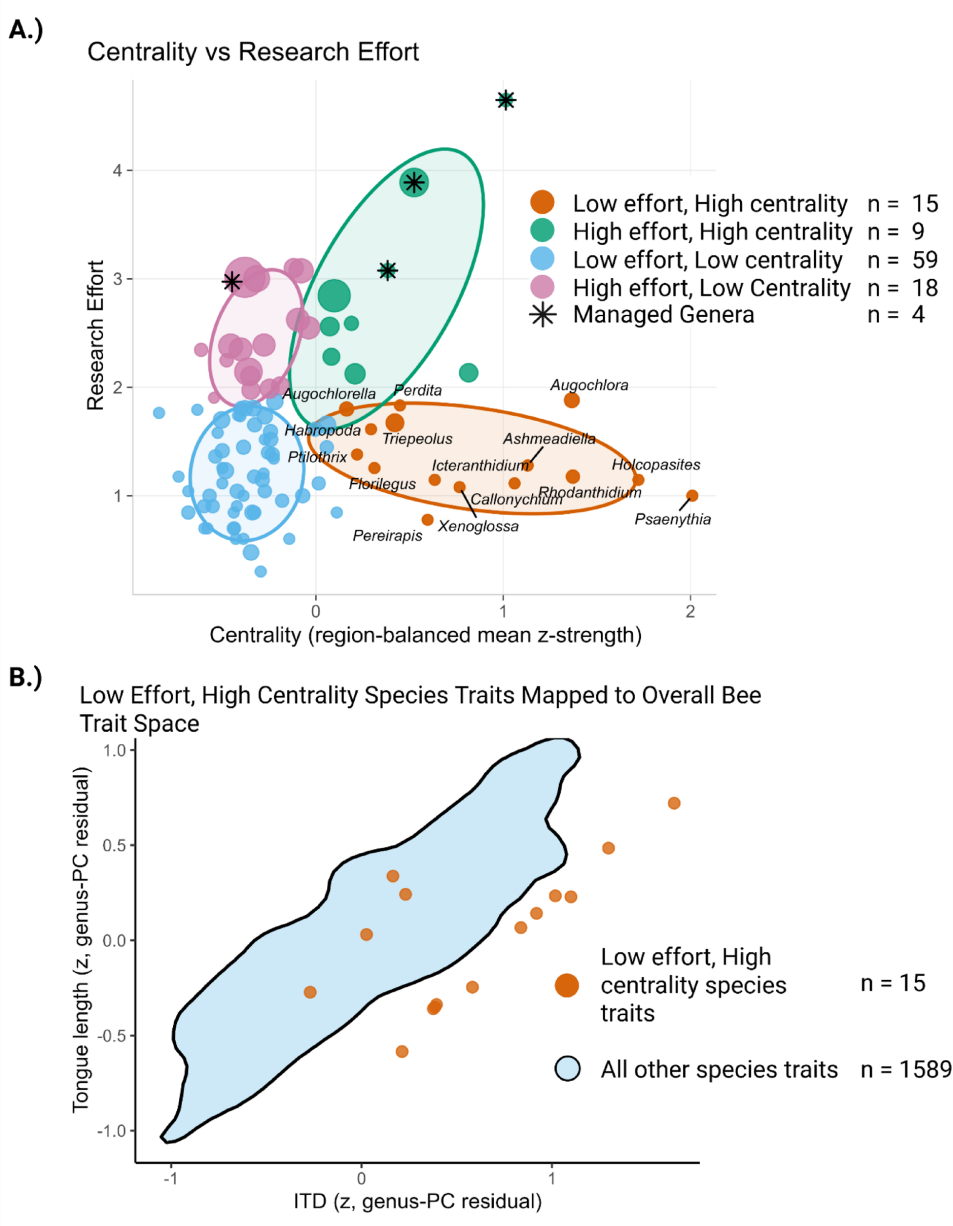
Centrality–effort mismatch, trait–space gaps, and modelled drivers of research counts. (**A**) Genus-level ecological centrality (x-axis; region-balanced mean z-strength from plant–pollinator interaction (visitation) networks) plotted against research effort. Points are genera; *marks managed genera. Coloured 68% ellipses summarise four ‘policy’ groups defined by effort (high/low) × centrality (high/low). Many genera fall into the high-centrality, low-effort quadrant, indicating high-centrality, low-effort targets (in recorded visitation networks). (**B**) Trait space of species: positions of species from the Low Effort, High Centrality (orange points) over the background envelope of all other species (teal, kernel-density isopleth). Axes are genus-PC–residualised z-scores for intertegular distance (ITD) and tongue length, showing that priority species occupy distinct portions of trait space not well covered by the broader literature. See Supplementary Fig. 2 for the rest of the classified species in trait space.

Research effort is decoupled from plant-pollinator visitation network centrality; the genera that are most central (highest species strength) in recorded visitation (interaction) networks are not the bees being studied the most. In the policy-group clustering of genera (Fig. 1A), most taxa fall into Low Effort, Low Centrality (LELC; *n* = 59), but distinct minorities of genera are Low Effort, High Centrality (LEHC; *n* = 15), High Effort, Low Centrality (HELC; *n* = 18) and High Effort, High Centrality (HEHC; *n* = 9). Managed genera are concentrated in the high-effort groups (managed share: 0.333 in HEHC; 0.056 in HELC) and are completely absent from both low-effort groups (managed share = 0 in both LEHC and LELC), underscoring that the genera attracting the most papers are not those that uniquely anchor network centrality. The scatter confirms that centrality and effort are not aligned (Spearman ρ = 0.140, *p* = 0.14). This weak association is robust to geographic sampling: excluding networks from any single world region yields similarly weak rank correlations (leave-one-region-out; Supplementary Table 4). Taken together, Fig. 1A identifies a set of 15 understudied genera that are disproportionately central in pollinator interaction networks but poorly represented in the literature, including *Psaenythia*, *Holcopasites*, *Rhodanthidium*, *Callonychium*, *Xenoglossa*, *Augochlora*, and *Ashmeadiella*. See Supplementary Data Tables 1, 2, 3 and 4 for all species assignments. LEHC genera identify under-studied but network central lineages with distinct trait profiles. ﻿LEHC (low effort, high centrality) genera identify under-studied but central lineages. The LEHC set comprises fifteen genera (101 total genera in the plot), and the species for which we have trait data from these genera are few (15 species; Fig. 1B). When those LEHC species are projected into two-trait space (Intertegular distance (ITD) and tongue length, N = 1,678 species total), they occupy a largely distinct trait space, with higher ITD on average. This trait space separation indicates that research gaps extend beyond individual taxonomic lineages to encompass entire functional groups, with implications for predictive ecology and functional diversity assessments.

Hypervolume analysis reveals that research effort clusters occupy less trait space than expected by chance across nearly all groups. LEHC genera cover 75.80% of overall trait space compared to a null expectation of 89.47% (*p* = 0.03), confirming these under-studied but network-central lineages occupy a distinct functional niche. Critically, even HELC genera, the most intensively studied cluster with 1,095 species, cover significantly less trait space than random sampling would predict (88.72% vs. 94.65% null, *p* < 0.001). LELC genera show similar concentration (76.04% vs. 90.15% null, *p* < 0.001). Only HEHC genera approach null expectations (93.07% vs. 94.20%, *p* = 0.21), though they do not exceed them. Managed genera show the most pronounced concentration, covering just 47.42% of trait space against a 70.43% null expectation (*p* < 0.001), indicating that commercial use targets a narrow functional subset of bee diversity.

Trait coverage is limited overall and biased toward managed bees. Across the known bee species, just 7.78% have measurements for the focal traits; coverage is 7.75% for wild species but 35.7% among managed species. That bias also changes what their trait space looks like: kernel-density hypervolume joins show reduced overlap between managed and wild species compared to chance expectation (overlap mass = 0.359 vs. 0.577 null, *p* < 0.001), indicating that managed bees occupy a functionally distinct subset of trait space rather than a representative sample. Wild-only space is substantial (unique-wild mass = 0.790) and managed-only space likewise occupies a distinct region (unique-managed mass = 0.570), though neither departs significantly from null expectations (both *p* = 0.89). Centroids for managed vs. wild are only weakly separated (0.067 vs. 0.168 null, *p* = 0.83).

**Fig. 2:**
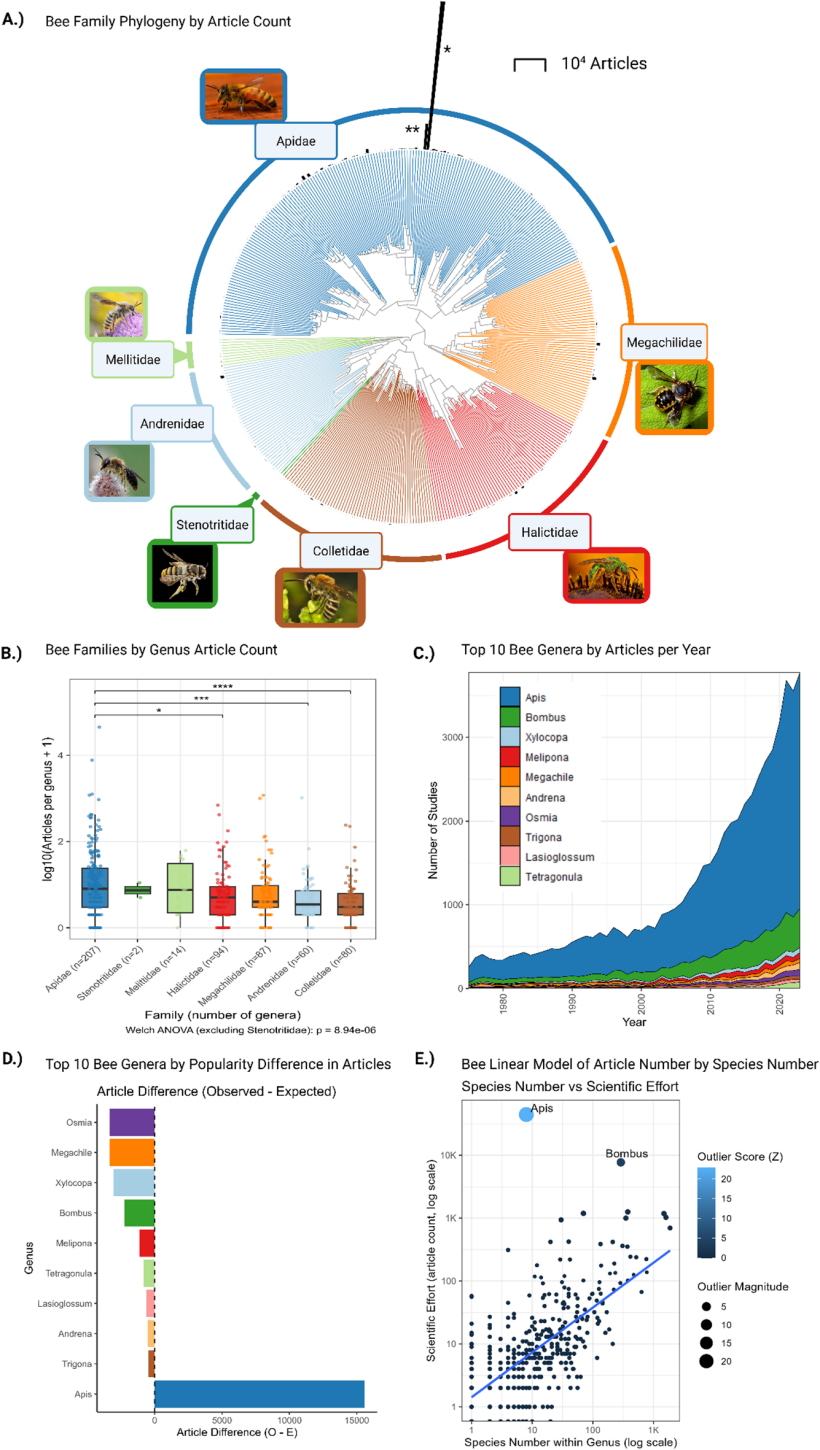
Global research effort across bee genera and families. (**A**) Circular phylogeny of bee genera with an outer radial bar for each tip showing total publications per genus (machine-learning–assisted counts). Tip colours indicate family; thicker coloured arcs label the major families. Bar lengths are in raw counts (scale bar = 10⁴ articles). * is *Apis* and ** is *Bombus*. (**B**) Distribution of papers per genus by family on a log₁₀(articles + 1) scale; points are genera and boxes show median and IQR. Welch ANOVA detected differences among families (F₅,₁₀₆.₃ = 7.12, *p* < 0.001). Games–Howell post hoc tests (BH-adjusted) identified three significant contrasts: Apidae had fewer papers per genus than Halictidae (Δ = − 0.260 log₁₀ units, *p*_adj = 0.02), Andrenidae (Δ = − 0.358, *p*_adj < 0.001), and Colletidae (Δ = − 0.431, *p*_adj < 0.001); all other pairwise contrasts were not significant. (**C**) Concentration of research effort through time (1975–2023). Stacked areas show annual counts as number of papers per year by genus. (**D**) Popularity-normalised bias in research effort. Bars show difference totals per genus (coloured) residuals (Observed − Expected). (**E**) Species richness vs. research effort with outliers highlighted. The fitted linear model (solid line; 95% CI band) yields a scaling exponent = 0.673 (95% CI 0.614–0.732, adj. R² = 0.512, robust s.e.). *Apis* and *Bombus* (labelled) lie far above the trend, indicating excess attention beyond biodiversity alone.

### Bee research is disproportionately concentrated on a small number of genera, especially *Apis* and *Bombus*, relative to overall bee biodiversity and public popularity

Across bee families using our validated machine-learning–assisted counts of papers, research effort remains highly uneven: a few genera dominate while most have few or none (Fig. 2A). Median (raw) research papers (or research effort) per genus were low in every family: Apidae 7, Halictidae 4, Megachilidae 3, Andrenidae 2.5, Colletidae 2, and Melittidae 6.5 (Stenotritidae has only 2 genera and was excluded from formal comparisons). On the log scale, per-genus research effort differed modestly (Welch ANOVA, F (5, 106) = 7.12, *p* < 0.0001). Games–Howell post-hoc tests with BH correction identified three significant contrasts, all involving Apidae having lower per-genus research effort than Halictidae (p_adj = 0.02), Andrenidae (p_adj *p* < 0.0001), and Colletidae (p_adj *p* < 0.0001); all other contrasts were non-significant (Fig. 2B).

Global output on bee genera rose steeply from 252 papers in 1975 to 3,574 in 2023, yet this expansion did not diversify attention across genera (Fig. 2C). Instead, research remained highly concentrated: *Apis* alone accounted for 63.8–76% of all top 10 most studied genus papers every year since 1975 (e.g., 71.8% in 1975; 74.5% in 2023), while *Bombus* contributed a further ≈ 9–16% annually (e.g., 9.8% in 1975; 12.4% in 2023). Together, *Apis* and *Bombus* represented ≈ 81% of the top-genus literature in 1975 and ≈ 87% by 2023 (Fig. 2C). Other widely distributed genera rarely exceeded 5% in any given year.

Public popularity-scaled expectations confirm a strong over-representation of *Apis* and broad under-representation of other highly studied genera (Fig. 2D). Using a baseline proportional to public interest (raw popularity) to compute expected paper counts across the ten focal genera in this analysis (*Andrena*, *Apis*, *Bombus*, *Lasioglossum*, *Megachile*, *Melipona*, *Osmia*, *Tetragonula*, *Trigona*, and *Xylocopa*), *Apis* accrued 44,431 papers versus 28,860 expected (+ 15,571; +54%). All other genera were under-represented relative to public interest, including *Bombus* (7720 vs. 9970; − 2,250; −23%), *Megachile* (1184 vs. 4522; − 3338; − 74%), *Osmia* (1001 vs. 4343; − 3342; − 77%), *Xylocopa* (1256 vs. 4294; −3038; − 71%), *Melipona* (1190 vs. 2,332; −1142; − 49%), *Andrena* (1030 vs. 1560; − 530; − 34%), *Lasioglossum* (697 vs. 1331; − 634; − 48%), *Tetragonula* (423 vs. 1259; − 836; −66%), and *Trigona* (939 vs. 1402; − 463; −33%).

Concentration persists even when accounting for biodiversity. Research effort (log articles + 1) against genus species richness yielded a slope of 0.673 (95% CI 0.614–0.732; adj. R² = 0.512; robust s.e.), indicating that publications increase slower than richness. There are fewer articles than species in a genus for the majority of species. The slope was nearly unchanged after removing high-influence (0.583, 0.531–0.635; 56 of 544 points removed; adj. R² = 0.475) and was similar when *Apis* and *Bombus* were excluded (0.660, 0.604–0.716; adj. R² = 0.543), demonstrating that the overall richness–effort relationship is robust to dominant genera. Results were similar when removing the top 1% by richness (0.658, 0.594–0.722) or by article count (0.633, 0.579–0.686). Despite this, *Apis* and *Bombus* are far above the richness trendline (Fig. 2E), reinforcing that the scientific research interest in them is not driven by their biodiversity. See Supplementary Fig. 3. for an alternative fit for richness–effort scaling.

### Management and life history structure bee research effort independent of *Apis* and *Bombus*

**Fig. 3:**
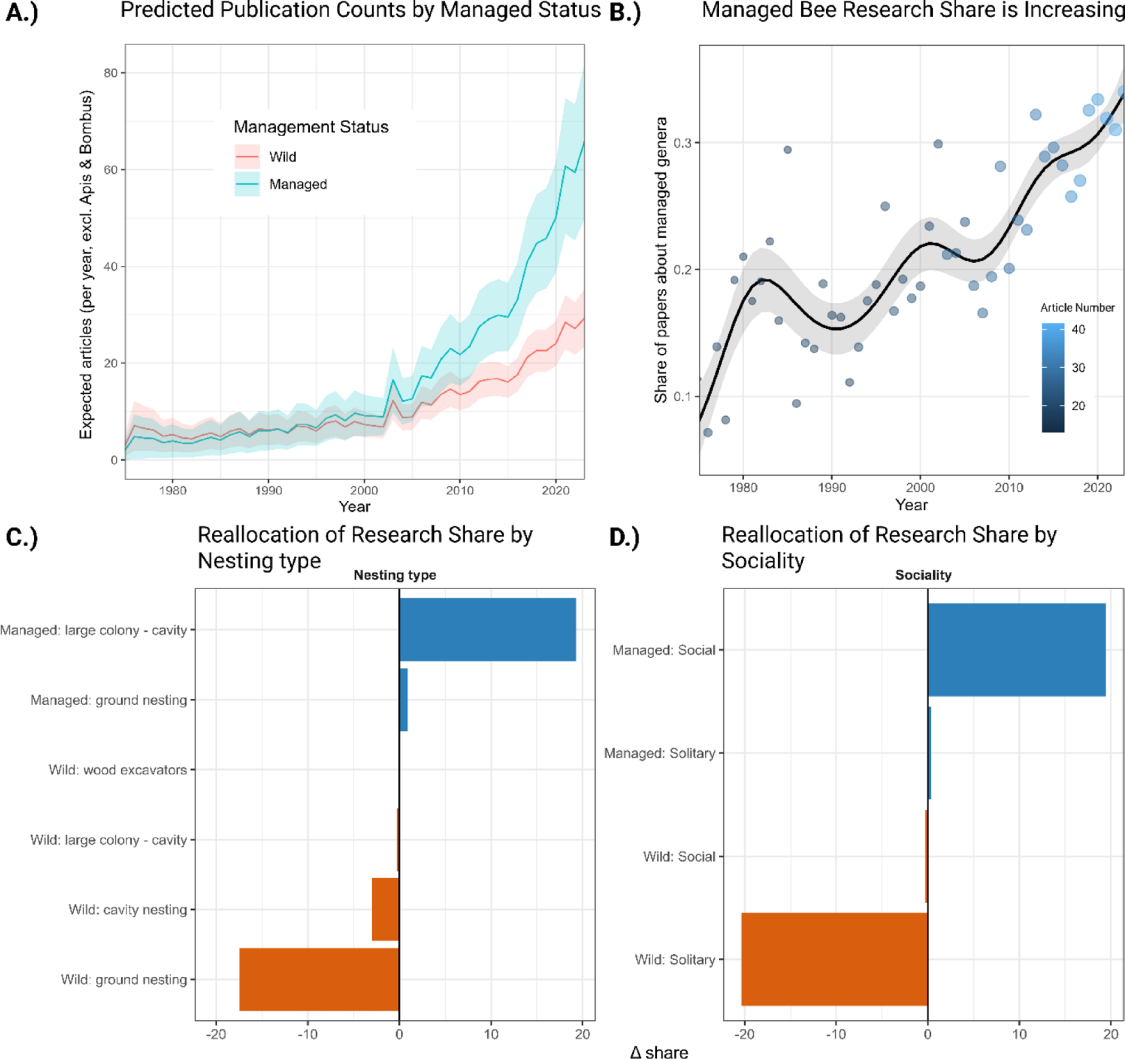
Bias through time after removing *Apis* and *Bombus* (**A**) Predicted annual paper counts for managed and wild genera. Managed genera have consistently higher expected counts that diverge over time, consistent with IRR = 2.86 and a positive Managed × Year interaction of IRR = 1.78. (**B**) Share of research about managed genera through time with points sized by annual volume and a fitted smooth. (**C**) and (**D**) Reallocation of attention summarised as percentage-point changes from early to recent windows for Managed or Wild split by Sociality and by Nesting type by Sociality. Gains concentrate in Managed: Social and Managed: large-colony categories, with losses in Wild: Solitary and Wild: ground or cavity groups. See Supplementary Fig. 4 for managed-bee premium over time separated into time frames.

Bee research effort is strongly structured by management status, sociality, and nesting ecology rather than randomly distributed across genera. *Apis* and *Bombus* are exceptionally dominant in the literature and inflate the managed premium or the ratio of research attention received by managed versus wild genera after controlling for species richness in the full dataset. With them included, managed genera receive 4.37 times more papers than wild genera (IRR 4.37, 95% CI 3.07–6.24, *p* < 0.001). Removing *Apis* and *Bombus* reduces but does not remove this pattern: managed genera still receive 2.86 times more papers (IRR 2.86, 2.10–3.89, *p* < 0.001), and the excess of research on managed bees (or managed premium) grows through time (Managed × Year IRR 1.78 per SD(year), 1.58–1.99, *p* < 0.001; Fig. 3A). To account for the overall exponential growth in scientific publishing, all count models use the log of total bee-genus papers in each year as an exposure offset, meaning reported effects represent changes in share or relative attention rather than absolute counts.

Removing *Apis* and *Bombus* in a noAB (no *Apis* or *Bombus*) count model shows additional, quantifiable structure. Species richness strongly predicts effort (IRR 1.50, 1.41–1.59, *p* < 0.001). Wood-excavating genera are favoured (IRR 1.92, 1.23–2.99, *p* = 0.004), whereas ground-nesting and large-colony cavity categories show no detectable main effect on counts after controlling for other factors (IRR 0.91, 0.75–1.27, *p* = 0.373 and IRR 1.09, 0.53–2.24, *p* = 0.812). Time-series share models confirm a field-level reweighting away from wild taxa and toward managed lineages even without *Apis* and *Bombus* (Fig. 3C and D). The managed share increases sharply over time (GLM slope β = + 0.0259 yr⁻¹, *p* < 0.001; GAM adj. R² = 0.747, *p* < 0.001; Fig. 3B). Social taxa gain share in parallel (β = + 0.0246yr⁻¹, *p* < 0.001) while solitary taxa decline (β = − 0.0246 yr⁻¹, *p* < 0.001). Nesting composition shifts toward large-colony cavity groups (β = +0.0235 yr⁻¹, *p* < 0.001) with declines for ground nesters (β = − 0.0123 yr⁻¹, *p* < 0.001), cavity nesters (β = − 0.00528 yr⁻¹, *p* < 0.001; the trend for wood excavators is small and non-significant (β = − 0.00228 yr⁻¹, *p* = 0.236) (see Supplementary Data Fig. 5).

Across all analyses, including and excluding *Apis* and *Bombus*, the direction of effects is consistent. Management status is the primary driver of bias, with social lineages systematically favoured over solitary ones, while nesting strategies also influence research attention. Removing *Apis* and *Bombus* lowers the absolute magnitude of the managed premium but reveals that the shift toward managed, social, large-colony lineages is a field-wide pattern rather than solely an artifact of two genera. This goes beyond the simple change in overall research output, see Supplementary Data Fig. 4 for a time series analysis of management premium over the overall increase in research output.

### Coverage clusters around managed taxa; trait gaps limit scope

**Fig. 4:**
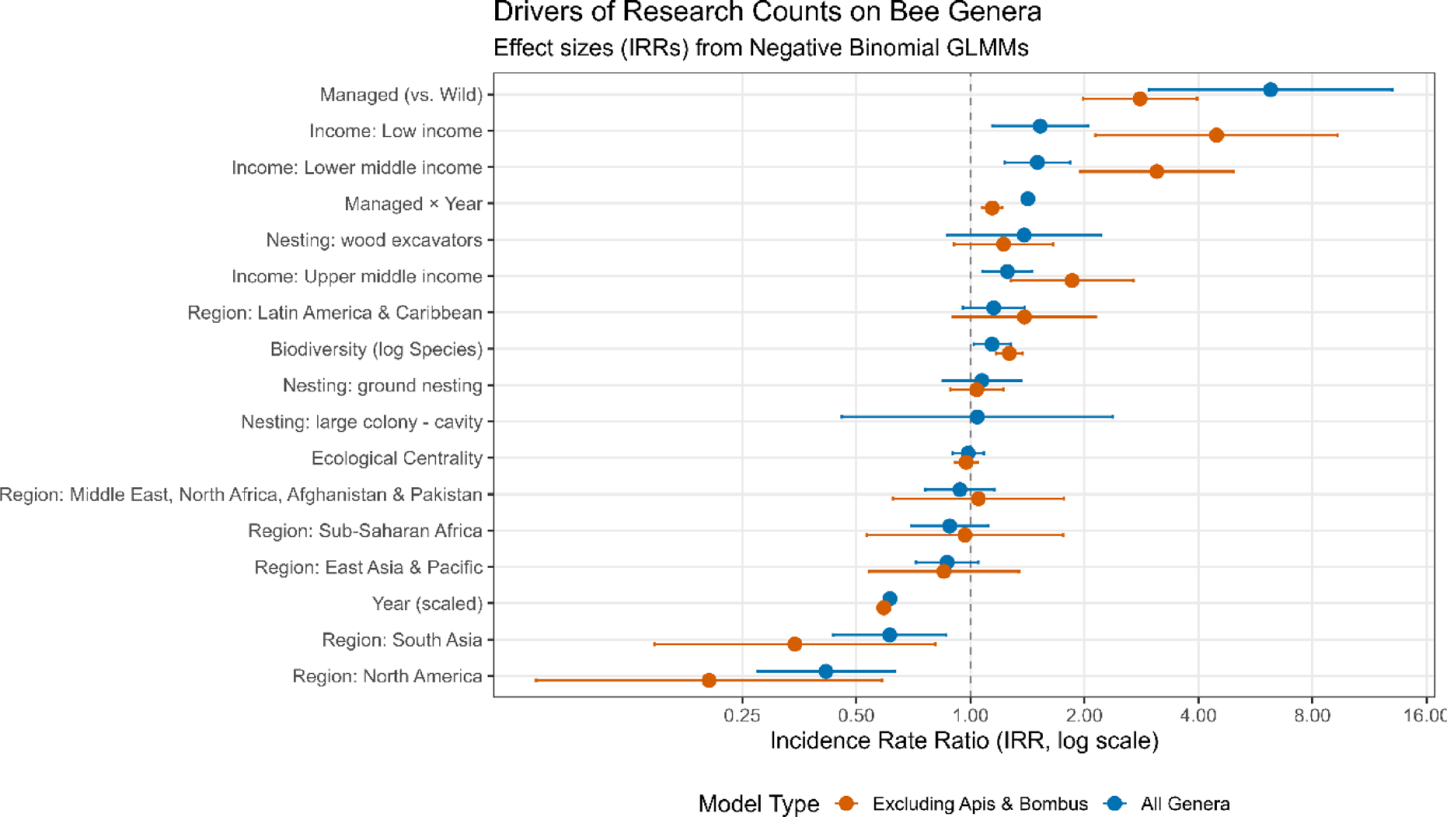
Determinants of yearly article counts from negative-binomial GLMM. Shown as incidence rate ratios (IRR; points) with 95% CIs (bars) on a log scale; dashed line = IRR=1. Blue: all genera; orange: excluding *Apis* and *Bombus*. Predictors include Managed (vs. Wild), Managed*Year, Biodiversity (log species), Ecological centrality (z-strength), Nesting contrasts (baseline = large-colony cavity), income group (baseline = High income), and World Bank region (baseline = Europe & Central Asia).

Article counts are driven by management status, macro-context, and nesting type rather than centrality (Fig. 4). In the integrated negative-binomial models (country–genus panel, with random effects for countries and genera and an offset for national output), managed genera receive markedly more papers than wild genera (All-genera IRR = 6.20, 95% CI 2.96–13.00, *p* < 0.001; excluding *Apis* and *Bombus* IRR = 2.81, 1.99–3.97, *p* < 0.001). Publishing intensity declines through time after controls (Year, scaled: IRR = 0.614, 0.598–0.629, *p* < 0.001), but the managed premium grows (Managed × Year: All-genera IRR = 1.419, 1.377–1.462, *p* < 0.001; excluding *Apis* and *Bombus* IRR = 1.142, 1.073–1.216, *p* < 0.001). Emphasis varies strongly among countries and increases over time; adjusted income/region contrasts and income-transition specifications are reported in Supplementary Results (Supplementary Data Fig. 1A–D). Countries with greater within-panel bee richness publish more per genus (All-genera IRR = 1.141, 1.020–1.277, *p* = 0.021; excluding *Apis* and *Bombus* IRR = 1.267, 1.170–1.373, *p* < 0.001). Geographic and income contrasts persist after traits and management are controlled: North America is below the global baseline (IRR = 0.416, 0.274–0.631, *p* < 0.001; excluding *Apis* and *Bombus* IRR = 0.204, 0.071–0.585, *p* < 0.001), while South Asia is also lower (IRR = 0.613, 0.435–0.864, *p* = 0.0052; excluding *Apis* and *Bombus* IRR = 0.344, 0.147–0.808, *p* = 0.014). Nesting effects are not statistically significant in this specification (Ground-nesting IRR = 1.073, 0.845–1.361, *p* = 0.564; Wood-excavators IRR = 1.387, 0.869–2.213, *p* = 0.170). In the corresponding models that include ecological centrality, the centrality term is small and non-significant once these covariates are included (All-genera IRR = 0.987, 0.897–1.086, *p* = 0.792; excluding *Apis* and *Bombus* IRR = 0.975, 0.909–1.046, *p* = 0.481), i.e., network importance does not explain where the literature is dense. See Supplementary Results: VIF analysis for collinearity analysis.

Overall, research effort clusters where taxa are already well covered and often managed, not where network centrality indicates the largest ecological leverage; the few LEHC species we can place fall toward the edge of trait space; and the global distribution of articles is shaped by management, macro-richness, regions, and nesting type rather than by centrality. These results are robust to removing *Apis* and *Bombus* and to alternative availability metrics, but they are constrained by trait data scarcity (only ≈ 8% of species have measurements) and by the small LEHC trait sample.

## Discussion

Global bee research effort is decoupled from plant-pollinator network centrality and morphological traits. The genera most central in pollination networks are not those most represented in the literature, and research concentrates on a functionally narrow portion of trait space. Overall, whether a bee genus contains human managed species is the clearest correlate of research attention. These managed genera have received far more papers than wild genera in negative-binomial models that control for traits and macro-context. This over representation has grown, and is still growing, over time. Research effort correlates positively with the number of species in a genus, while the effect of ecological centrality is weak once other factors are controlled for. These patterns hold in country–genus panels with random effects for countries and genera and an output offset. Traits and networks add new ecological structure. Centrality and effort are only weakly aligned at the genus level, producing a compact set of low-effort, high-strength priorities that are poorly represented in the literature. Trait coverage is thin overall and biased toward managed lineages: about 7.8% of species have the focal measurements, coverage is about 35.7% among managed species, and managed–wild hypervolumes overlap modestly with a large wild-only region. This decoupling becomes clearer when genera are grouped by the joint distribution of effort and centrality. Managed genera are concentrated in the high-effort groups and are absent from both low-effort groups (managed share = 0 in LEHC and LELC), underscoring that management is strongly coupled to attention but not uniquely to network importance.

Trait-space results indicate that these gaps are functional as well as taxonomic. When the LEHC species that can be placed in two-trait space (intertegular distance and tongue length) are projected against the broader species envelope, they occupy a distinct region, offset from species with well-sampled trait combinations. Hypervolume tests reinforce that effort clusters generally span less trait space than expected under null sampling: LEHC, HELC, and LELC cover a smaller percentage of overall trait space than expected. Only HEHC approaches null expectation. Consistent with this, trait coverage is thin overall and biased toward managed lineages: ~7.8% of bee species have the focal measurements, but coverage is ~ 35.7% among managed species. Managed–wild hypervolume overlap is modest and lower than expected by chance, with a large wild-only region. Thus, focusing on managed taxa captures a functionally non-representative subset of bee diversity: centroids are not strongly separated, but the span of trait space is, leaving substantial wild functional space poorly characterised.

The field has reweighted research effort toward managed bees even when excluding *Apis* and *Bombus*. The share of publications on managed bees rises from about 10% in early years to about 31% in recent years, with parallel gains for social, large colony, cavity lineages and declines for solitary, ground nesting groups. These trajectories are consistent with the growing managed premium in the count models and with region and income effects that persist after controls. Biodiversity alone does not explain where effort concentrates. Publications overall scale sublinearly with genus richness, with slopes of about 0.68, and *Apis* and *Bombus* sit far above this trend. Popularity accounts for dominance among a few highly visible genera, but it does not recover the many network central, low effort genera that remain understudied. Together, these results indicate structural preferences that concentrate evidence on a narrow section of bee diversity, intensifying over time, likely driven by management, economic importance, methodological convenience, and cultural prominence^17^.

Multiple feedbacks reinforce this focus on managed and social bees. First, *Apis mellifera*, *Bombus terrestris*, and *Bombus impatiens* are major commercial pollinators, attracting public and private funding to safeguard their viability (Supplementary Table 2)^49–52^. Second, these taxa are culturally prominent and charismatic^53,54^. Third, they are tractable experimental systems suitable for standardised toxicological and parasitological work (Supplementary Table 3), but not necessarily indicative of the majority of other solitary bees^55–57^.

Our corpus includes all bee-related publications regardless of research domain. Studies using honey bees or bumblebees as model organisms for molecular biology or immunology are retained, which may inflate *Apis* and *Bombus* counts. However, this inclusion is appropriate because our question concerns research attention distribution, not just pollination ecology, and model organism status itself reflects the research bias we document. Our inferences are limited by an English, indexed corpus; title/abstract–based extraction; genus-level proxies (e.g., popularity by the most-studied species); and uneven regional network coverage. Many high-centrality genera appear in few networks, and genus-level analyses are observational, so coefficients are descriptive rather than causal. Category overlaps and sparse trait coverage further reduce sensitivity, but these are common limitations of large-scale ecological network studies^58–61^. Network centrality is influenced by species abundance and sampling intensity, so genera that appear central may do so partly because they are locally common^62^ and frequently encountered. Our centrality metric should therefore be interpreted as reflecting structural position in recorded networks rather than unique pollination capacity. Despite these constraints, network approaches remain useful for revealing broad-scale structure, identifying consistently central taxa, and generating hypotheses for targeted investigation.

This research bias has tangible ramifications for understanding pollinator decline. Numerous studies document declining trends in global insect populations, including solitary bees^63–65^. Solitary bee populations in Britain, for instance, have declined by 32% since 1980, while managed honey bees and bumblebees actually increased over the same period^64^. Additionally, historical declines in honey bee colonies including a 25% decrease in Europe between 1985 and 2005^14^ and 59% decrease in North America between 1947 and 2005^66,67^ have galvanised public concern but not translated into proportionate attention for the wild species that often face equal or greater risks. Although the ‘charisma’ of honey bees does increase general awareness about pollinators, it can obscure the diversity of other bee species and thus skew conservation messaging^23,24^. Our popularity index analysis confirms that even adjusting for public interest, *Apis* far exceeds the expected number of publications by ≈ 15,000 articles, further demonstrating the strength of economic and cultural factors in shaping research agendas. Shifting public narratives to include solitary and ground-nesting species could spur a broader research focus and better align conservation efforts with ecological reality. Basic data on population trends are often lacking. For instance, the IUCN Red List states that across Europe 9.2% of bee species (9.1% in the EU-27) are already threatened with extinction, another 5.2–5.4% are considered ‘Near Threatened’, but the truly critical figure is that more than half of all recorded species (1,101 in Europe and 1,048 in the EU-27) are classified as ‘Data Deficient’, meaning we currently have no idea how secure they are^68^.

Addressing this bias necessitates a multifaceted approach. We recommend researchers and funding agencies strive to diversify research portfolios, avoid relying on a single model species to represent all bees, and increase attention to solitary and underrepresented taxa to better capture the complexity, adaptability, and vulnerability of bee communities. A central barrier is the lack of basic trait and interaction data, since only about 8% of species have measurements for core traits, coverage is much higher for managed species than for wild species, and pollination network data are sparse and uneven across regions and genera. We also suggest using phylogenetic distance as an extension for traits as a valuable direction for future research. Mechanisms that incentivise study of non-model organisms, through targeted funding calls and institutional support, should explicitly require collection and open deposition of trait and network data using standardised protocols. Integrative frameworks that combine citizen science, museum collections, genomic databases, and long-term monitoring programmes can illuminate understudied species and their interaction structure, and should prioritise trait measurement and network sampling in data-poor lineages and locations to improve inference on population trends, ecological roles, and vulnerability to environmental change^17,69,70^. Priorities include coordinated trait campaigns on wild species for key traits such as body size and tongue length, expansion of interaction monitoring across seasons and habitats, linkage of vouchers to sequences and trait records, and capacity building in under-sampled regions.

Ironically, we must acknowledge that this paper only considers bees, but insect pollinators are found across the insect tree of life. Thus, ultimately, a shift toward more inclusive insect pollinator research would foster a stronger foundation for the conservation of whole ecological networks (or at least plant-pollinator mutualisms), ensuring that efforts to mitigate declines address the breadth of biological diversity. The corpus, alias-library approach, and network/trait workflow generalise readily to other threatened groups, while trait standards may need adaptation; we offer these components so the community can audit and optimise alternate research portfolios. Rather than a deficit narrative, we frame this as optimisation under uncertainty: diversify research effort across taxa, traits, and geographies to improve understanding of pollination services and ecological resilience. By understanding research imbalances, the scientific community can better safeguard global pollination services critical for biodiversity, ecosystem function, and food security^71,72^.

## Methods

**Fig. 5:**
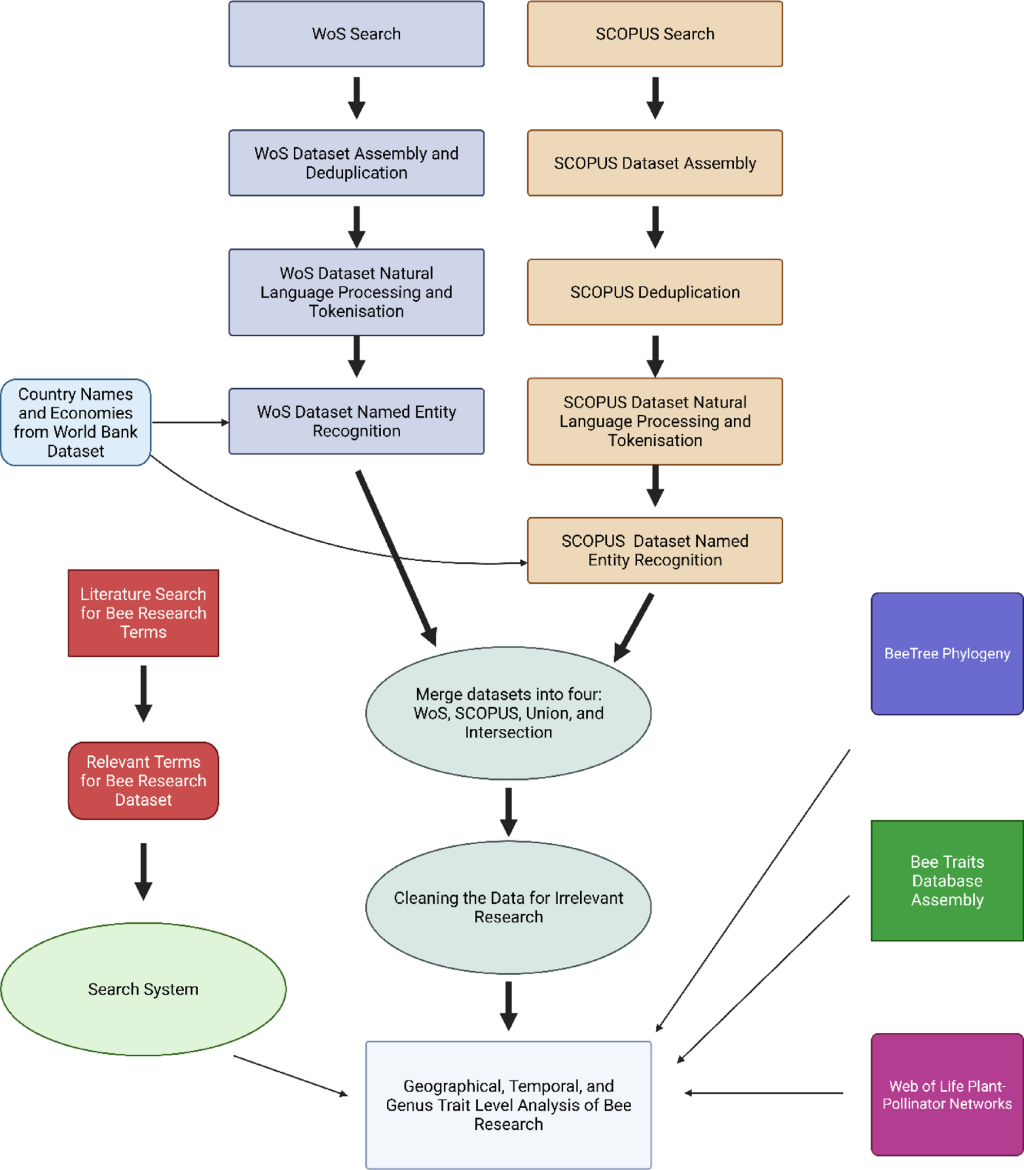
Workflow for machine learning. A graphical representation of the methods used in this analysis. It details the parallel searches for WoS and Scopus, data deduplication, NLP processing, NER processing, dataset curation and cleaning, and the search system.

This study used an automated text-mining pipeline^73^ to search for, process, and extract data on bee genera from 106,781 publications sourced from Web of Science (WoS) and Scopus queried 1950–2024; after filtering and completeness constraints, analyses use 69,682 publications from 1975 to 2023. This publication data was then merged with datasets on genus traits, geography, and public interest to statistically analyse research biases across time, regions, and traits (Fig. 5.). Data curation and machine learning were performed in Python version 3.10. We analysed the corpus of bee research in R (v4.2+)^74^. Data wrangling and visualisation used the tidyverse^75^ meta-package, including ggplot2^76^, dplyr^77^, tidyr^78^, forcats^79^ and stringr^80^; figures were composed with cowplot and patchwork^81,82^, with non-overlapping labels via ggrepel^83^. Plot utilities and palettes used scales, viridis, ggpubr, and ggforce^84–87^. Model diagnostics and marginal means used DHARMa and emmeans^88,89^. Project paths and parallel backends used here and doParallel^90,91^.

### Data collection

We first queried WoS collections (Core Collection; CABI: CAB Abstracts^®^; BIOSIS Citation Index; MEDLINE^®^; SciELO; KCI) and Scopus for 1950–2024 using equivalent bee-focused strings (See Supplementary Table 1. for search strings). We restricted both to English-language documents and to the following types: Article, Review Article, and Dissertation/Thesis; exports included identifiers (UT for WoS, EID for Scopus), DOI, title, abstract, year, document type, language, affiliations, and country fields [WoS; Scopus]. A paper that mentions multiple genera contributes one count to each mentioned genus, but each paper is counted at most once per genus. We do not trace citations; reviews are distinct research effort, and theses are inconsistently published so dataset-level deduplication is infeasible and risks undercounting less visible genera. For each source, we cleaned in parallel: headers to lower case; character fields trimmed; publication year coerced to integer; and DOIs normalised by lower-casing and removing doi: prefixes. Within-source duplicates were removed by keeping the first record per UT (WoS) or EID (Scopus) and dropping residual duplicates sharing a normalised DOI. This produced 91,797 unique WoS records (initial 92,889) and 63,871 unique Scopus records (initial 65,431). See Supplementary Table 1. for detailed search queries.

### Machine learning pipeline for article counts by genera and validation of the dataset

After cleaning each source separately, we harmonised Web of Science and Scopus and de-duplicated them using a two-step, precision-first matcher. First, we did an exact merge on normalised DOIs. For records without DOIs or with inconsistencies, we used a title–year approach: remaining items were grouped by publication year and compared with a token-overlap string similarity method^92^, accepting only very high-confidence matches. The matched set defined the intersection (49,066 records); the remainder formed WoS-only (42,731) and Scopus-only (14,984) sets and together yielded 106,781 unique documents. For intersected records, we kept a source flag indicating provenance.

For natural language processing, we used spaCy^93^ with its transformer-based English pipeline to process titles and abstracts in batches, producing tokens, parts of speech, base forms of words, and noun phrases. Tokens, individual words or word-pieces used as input units for the language model, were normalised (lowercased, punctuation removed) and we applied Porter stemming (reducing words to their root form, e.g., ‘pollinating’ → ‘pollin’) to both search queries and target text to improve matching across morphological variants.

We then ran named-entity recognition with the same spaCy pipeline to extract geographic mentions (countries/locations)^93^. These were linked to the World Bank’s catalogue of economies using a curated dictionary of canonical names and common historical/alternative forms^94^. We prioritised exact matches and dropped uncertain fuzzy matches to keep geographic assignment conservative.

To focus on bee-relevant articles, we applied a topic-modelling workflow (BERTopic) that uses sentence-level embeddings to capture meaning, followed by dimensionality reduction and density-based clustering to discover themes^95–98^. As a conservative filter, we retained the main, central ‘bee’ topic and unassigned outliers and excluded peripheral topics likely to reflect tangential content. The search system operated on a unified table of parsed document tokens, leveraging publication year metadata from both Scopus and WoS. The foundational component of our methodology was a Porter-stemmed inverted index (a lookup table that maps each stemmed term to the set of document IDs where it appears)^99^, which mapped each unique stemmed token to the set of document IDs in which it appeared. This index enabled efficient and deterministic matching against bee genera. A total of 544 bee genera from seven families were identified using Discover Life^100^, an interactive online encyclopaedia with an up-to-date Hymenoptera database (as of July 2024). Each entry includes the full number of species in each genus which totalled 20,759 species, corroborated by OneZoom^101^. To maximise recall, we applied a standardised pre-processing routine to each library. This involved dynamically identifying all relevant columns for alias generation, normalising the text by lowercasing and trimming whitespace, and splitting multi-valued entries on common delimiters. Furthermore, we automatically generated binomial abbreviations (e.g., ‘*A. mellifera*’) and incorporated manual common-name expansions not only for *Apis* (‘honey bee’ / ‘honeybee’) and *Bombus* (‘bumble bee’ / ‘bumblebee’) but also other unambiguous cases we used routinely in the literature, namely carpenter bee(s) are represented as *Xylocopa*, mason bee(s) are represented as *Osmia*, leafcutter / leaf-cutting bee(s) are represented as *Megachile*, and mining bee(s) are represented as *Andrena*. All resulting aliases were then Porter-stemmed before matching.

For the genus theme, this process yielded yearly counts for each specific genus, which were also aggregated by broad categories such as Sociality, Management, and Nesting Type again noted from Discover Life. Because our response is genus-level research effort, predictors must be genus-level. We intentionally use a conservative binary definition because sociality, and management status are properties of lineages that can disproportionately shape research attention through a small number of focal species. Treating mixed genera as ‘unmanaged’ (or ‘variously social’) would misclassify genera whose literature is dominated by a few focal species and would tend to make effects look smaller than they are. We therefore code ‘managed’ as a binary indicator for whether a genus contains any managed species; this captures whether the *presence* of a managed lineage can elevate research attention, even if many species in that genus are unmanaged (e.g., *Bombus*). We treated ‘large-colony cavity/ground’ as a separate category because key large-colony genera (notably *Apis* and *Bombus*) span multiple nesting contexts and are often reported inconsistently across sources. Sociality was classified following Discover Life into ‘Social’ (including eusocial genera such as *Apis* and *Bombus*, as well as primitively social genera) versus ‘Solitary’. We acknowledge that sociality exists along a spectrum^102^ and that our binary classification groups taxa with varying degrees of social organisation. We also calculated within-theme co-occurrence counts for pairs of genera and conducted a separate analysis to generate yearly counts for individual managed species. Finally, the genera library itself was annotated with the total article counts discovered for each entry. When country metadata were available, we also produced country-by-year versions of these counts.

To validate the automated counts, we performed two complementary assessments. First, we conducted comprehensive manual validation of 1,200 papers stratified by detection method to assess precision. Genus names achieved 90.0% precision (95% CI 86.1%–92.9%) and common names 93.0% precision (95% CI 89.5%–95.4%). Second, we compared machine-derived genus totals against manual reference counts that had been previously compiled from Web of Science for all 544 genera. Initial validation revealed lower-than-expected correlation (Spearman ρ = 0.83) due to imprecise handling of binomial abbreviations (e.g., ‘A. mellifera’) and epithet matching in the automated pipeline. We implemented a corrected matching algorithm that restricted automated counts to high-confidence full genus names and common names only, excluding ambiguous abbreviated forms. This improved agreement substantially (Spearman ρ = 0.928, 95% CI 0.909–0.944; Pearson *r* = 1.000, 95% CI 0.999–1.000), with mean absolute error of 49.1 papers per genus and root mean squared error of 486.9 papers. The linear relationship between manual and automated counts showed very strong fit (R² = 0.999, slope = 0.801, 95% CI 0.799–0.802), indicating the corrected pipeline accurately reproduced manually validated counts across the full range of research effort. The slope below 1.0 reflects conservative automated matching that prioritises precision over recall.

The final outputs included detailed yearly counts for all specific terms and broad categories, co-occurrence data and a high-level summary file tracking the total number of publications per year alongside the annual count of documents that mentioned any term from the bee genera; in addition we provided the annotated genera library, and a validation bundle comprising the detailed comparison, bucket summaries, outlier tables, bootstrap intervals and recall statistics.

### Centrality and trait-space analysis with respect to research effort

We quantified network roles using two metrics. First, we calculated species strength (the total dependence of all partners on a focal species) to identify taxa that disproportionately support/visit many plants^28,31^. Second, we estimated complementary specialisation (d′), which measures how uniquely a species uses a subset of available partners beyond what partner availability predicts, thereby highlighting taxa that contribute unique interactions^26^. To relate roles to functional traits, we included intertegular distance as a proxy for body size, which predicts flight capacity and foraging range and thus the spatial footprint of pollination and potential to bridge fragmented habitats^30,46^, and tongue length, which constrains floral accessibility and partitions niches among coexisting taxa, shaping both specialisation and complementarity^26,28^.

We derived network centrality from 176 (filtered down to 89 as detailed below) globally distributed plant-pollinator networks from Web of Life^103^, comprising both binary (*n* = 43) and weighted (*n* = 46) data types. To mitigate biases from network heterogeneity, we z-scored metrics within each network before averaging and computed region-balanced summaries. Sensitivity analyses stratifying by network type showed positive but weak relationships across network types, with the correlation stronger in binary networks than weighted networks (Supplementary Fig. 6). Bootstrap resampling (1000 iterations) confirmed that the centrality-effort correlation is robust [95% CI 0.04, 0.33]. We note that managed genera appear in substantially more networks than wild genera (median 25 vs. 2), indicating that sampling bias favours managed bees; centrality estimates for wild genera are therefore conservative. These are labelled plant-pollinator networks in the Web of Life metadata, but it may be more accurate to define them as plant-pollinator interaction networks. For each network we removed empty rows and columns, oriented pollinators on columns, parsed pollinator labels to Genus, and harmonised synonyms. We computed species strength and Blüthgen’s d′ using functions from the bipartite package, and when species strength was unavailable, we used column sums. If there were fewer than 3 matching pollinators we removed the network from the analysis. Within each network we z-scored metrics and stored the number of interactions for weighting. We linked networks to regions via the cited references and created a region‐balanced genus summary by averaging z‐scores within regions and then across regions when available, otherwise using an interaction‐weighted pooled summary^26,104^. As these networks record flower visitation rather than confirmed pollination events, our centrality metrics reflect structural importance in interaction networks, as a proxy for potential pollination effectiveness.

We tested alignment between centrality and research effort by joining region-balanced z‐strength to total per‐genus articles, computing a Spearman rank correlation, and plotting ranks with managed genera flagged. We grouped genera in the centrality–effort plane using quadrants at different thresholds to separate high vs. low effort and high vs. low centrality and exported the group assignments.

To characterise trait-space coverage we assembled species‐level trait data on 1678 species of bees from the Ostwald review^58^, BeeFunc^105^, Brazilian bee functional trait dataset^106^, and Safeguard^107^. We then mapped species to managed vs. wild and to the high‐centrality low‐effort set, and residualised intertegular distance and tongue length on genus principal components to remove genus-mean effects and reduce pseudoreplication from taxonomic clustering. We computed kernel‐density isopleths on a common grid and derived overlap and group‐unique space with permutation tests. As a complementary view we built two‐dimensional Gaussian hypervolumes for managed vs. wild and for policy groups and summarised overlap statistics. For Gaussian hypervolumes we used the hypervolume^108^ package’s automatic bandwidth estimator (estimate_bandwidth function; no manual tuning). For 2D kernel-density contours we used the default bandwidth of MASS kde2d, which applies a Scott/Silverman rule-of-thumb bandwidth selector.

### Research effort bias of biodiversity and popularity

Bee phylogenies were compiled from BeeTree^109^. Phylogenetic visualisations used ape, phytools, ggtree, ggtreeExtra, and ggnewscale^110–113^. To compare article counts among families, we analysed log-transformed totals to stabilise variance and retain zeros. Families with fewer than two genera with non-missing values were excluded from the inferential test set (Stenotritidae remained displayed in plots but was omitted from the inference where applicable – given that there are only two genera in this family). We used Welch’s heteroscedastic one-way ANOVA to test for differences among families, followed by Games–Howell pairwise comparisons; adjusted p-values were controlled using the Benjamini–Hochberg false discovery rate procedure^85,114–119^.

We summarised field growth with time-series of total annual publications by theme and by genus (absolute counts and proportions). We identified the top 10 genera by total articles and plotted stacked area series for 1975–2023^76,81^.

To quantify how research effort scales with biodiversity, we fitted a log–log linear model at the genus level adding 1 to accommodate zeros. We reported heteroskedasticity-robust (HC3) standard errors and 95% CIs and provided residual diagnostics (residuals–fitted and Q–Q)^120,121^. We flagged high-influence observations as Cook’s distance > 4/n or leverage > 2p/n and refit after excluding them^120,122–124^. Additional sensitivity trims dropped the top 1% of genera by species richness and, separately, by article count. For display we overlaid a LOESS smoother on the linear fit^125,126^.

To determine the relationship between public perception and scientific effort we used OneZoom^101^. OneZoom provides a ‘phylogenetically informed popularity score’ for nearly every described species by linking its comprehensive tree of life to the Wikimedia ecosystem. The system maps each taxon to its corresponding Wikipedia page and analyses page-view statistics to quantify public interest, effectively translating web traffic into a popularity metric. OneZoom takes mapping from OTT ID to Wikidata item (or ‘Q’) ID, which can be used to identify titles for taxon pages on Wikipedia; Wikimedia dump files can then be searched for statistics associated with these page titles^101^.

We used OneZoom’s popularity score as a proxy for public interest. Because popularity is often concentrated in a genus’s best-known species, we used the most popular species in each genus as the genus-level proxy (normalised to *Apis*). We then compared observed research effort to effort expected under a simple proportional relationship with popularity by computing an observed-to-expected ratio.

### Management and life history analysis of research effort

We constructed a genus–year table of article counts joined to traits for management status, sociality, nesting type, and species richness. From this we defined two analysis datasets: a full set that included *Apis* and *Bombus* and a noAB subset that excluded them. For each year we computed the total number of bee-genus papers and joined this as a denominator for proportion of research and as an exposure offset for count models.

To describe trajectories through time we modelled yearly proportion of research with binomial GLMs using a two-column response for successes and failures, and we produced a complementary sensitivity with a GAM that used a spline in year for the managed share only^127,128^. Sociality and nesting compositions were analysed in parallel with one-vs-rest binomial GLMs for each level, and p-values across levels were adjusted with the Benjamini–Hochberg procedure^114^. These share models were fitted once in the full dataset and again in the noAB subset.

To explain variation in article counts we fitted negative-binomial GLMMs via glmmTMB with a log link^129^. Fixed effects were management status, the interaction between management and scaled year, sociality, nesting type, and log1p-transformed species richness that was z-scaled. The random-effects structure included a genus intercept and a year slope by genus. We used the log of the total bee-genus papers in that year as an exposure offset. We estimated two variants in both the full and noAB datasets: a Top-10 model that included scaled log1p popularity where available, and a full-dataset model without popularity. We reported fixed effects as incidence rate ratios with Wald confidence intervals. Model diagnostics used DHARMa residual checks and standard overdispersion and zero-inflation assessments^88,130^.

Predicted population-level curves for managed versus wild and for social versus solitary were generated by setting continuous covariates to their sample means and marginalising over random effects. To summarise reallocation through time we compared early versus recent five-year means of the managed share in both the full and noAB datasets and expressed the change as percentage-point differences, alongside analogous summaries for sociality and nesting compositions.

The exposure offset controls for the exponential growth in overall publishing, ensuring that effects represent changes in the share of attention rather than absolute counts. As a robustness check, we also fit models separately for early (1975–1995), middle (1996–2010), and recent (2011–2023) periods to verify that effects are present within discrete temporal windows and not artifacts of comparing distant eras.

Inferential models were fitted with glmmTMB and mgcv^128,129^: count outcomes used negative binomial (nbinom2), and binary outcomes used a logit link. Continuous predictors noted as ‘scaled’ were standardised. Robust inference/diagnostics used broom.mixed, sandwich and lmtest^130–132^.

### Geography and income analysis of managed and social emphasis, with income-transition tests

We made world research maps with rnaturalearthdata with sf/rnaturalearth^133^. Network/ecological analyses also used vegan^134^. We constructed a country–genus–year panel, standardised country names to ISO3, and joined World Bank income group and region. Country and economic data was gathered from the World Bank Database^135^. In the World Development Indicators all 189 World Bank member countries, along with 28 others with populations of more than 30,000 are classified by income level and geographic region. Key classifications are geographic regions, income groups, and operational lending categories of the World Bank Group. For each country–year we computed total bee-genus papers for use as a denominator and as an offset^94^.

We estimated the country–year share of papers about managed genera with a binomial GLMM that included fixed effects for income group, region, and scaled year and a random intercept for country. The outcome used a two-column response for managed successes and non-managed failures. We reported odds ratios with Wald intervals. We fit an availability‐controlled variant that added a within‐dataset proxy for the fraction of present genera that are managed.

We modelled per-genus article counts within country–years with a negative‐binomial GLMM that included fixed effects for management, sociality grouped to social vs. solitary, scaled log1p species richness, income group, region, and scaled year. Random intercepts were specified for genus and country, and we used the log of total country–year bee‐genus papers as an exposure offset^129^.

### Final synthesis of research effort correlations

To evaluate whether centrality predicts research counts after traits and context we merged z-strength into the genus–year modelling frame and fitted negative‐binomial GLMMs with management, the interaction between management and scaled year, sociality, nesting type, scaled log1p species richness, and z‐strength as fixed effects. The random‐effects structure included a genus intercept and a year slope by genus, and we used the log of total year output as an exposure offset. We ran all‐genera and noAB variants and reported fixed effects as incidence rate ratios with Wald intervals^129^. We analyse English-language, indexed documents, so non-English and non-indexed work may be under-represented and coverage varies by region. Extraction operates on titles/abstracts; co-mentions need not indicate study focus, and some aliases are missed despite conservative curation. Popularity is proxied at the genus level by the most-studied species, which can blur within-genus heterogeneity. Network coverage is uneven across regions, and some high-centrality genera occur in few networks, so centrality estimates reflect the current archive rather than complete global coverage (Supplementary Fig. 6 demonstrates that the centrality–effort association is robust to alternative filtering rules). Analyses are observational at the genus level; coefficients represent associations, and genus-level categorisation can collapse taxa spanning multiple categories. Trait space is incompletely covered. As a robustness check, results are unchanged when *Apis* and *Bombus* are excluded.

## Supplementary Information

Below is the link to the electronic supplementary material.

Supplementary Material 1

## Data Availability

Code and derived, aggregated datasets are available at Zenodo: Nesbit, M. (2026). Mapping global bee research with traits and plant-pollinator interaction networks - code and data. Zenodo. DOI:(10.5281/zenodo.18680740). Licensed bibliographic corpora (e.g., Web of Science/SCOPUS) cannot be redistributed and are available from the original providers under their terms.
