# Supplementary material for: Mapping global bee research with traits and plant-pollinator interaction networks
Source: Sci Rep. 2026 Mar 10;16:12844. doi: 10.1038/s41598-026-41830-7 (PMC13096181; doi:10.1038/s41598-026-41830-7)
Supplement: Supplementary file 1 — Supplementary Material 1 [file 41598_2026_41830_MOESM1_ESM.pdf]

Mapping global bee research with traits and plant–pollinator interaction networks

Supplementary Materials

Miles Liam Nesbit[*1,2], Cecilia Montauban[1], Francis Windram[1], Miguel Santiago Bailey Pérez[1], William O.H. Hughes[3], Dave Goulson[3], Richard J. Gill[1], Peter Graystock[1]

[1]Georgina Mace Centre for the Living Planet, Department of Life Sciences, Imperial College London, Silwood Park, Ascot, Berkshire, United Kingdom

[2]The Grantham Institute, Imperial College London, South Kensington, London, United Kingdom

[3]School of Life Sciences, University of Sussex, Brighton, United Kingdom

[*]Corresponding author: Miles Liam Nesbit

m.nesbit19@imperial.ac.uk

Keywords: bees, pollinators, conservation, policy, plant–pollinator networks, trait analysis

Abstract

Bees sustain key functions in natural ecosystems and agricultural landscapes, yet our understanding of their ecology is typically informed from studies concentrated on a few model taxa. To reveal how this may be biasing our understanding of bee responses and function in the environment we quantify global patterns of research attention across 69,682 bee-related publications to test whether research effort aligns with plant-pollinator network centrality, trait variation, public interest, and socio-economic context. Human managed bees take up most of the research effort; importantly this trend has been increasing over time. Plant–pollinator network centrality is unrelated to research effort; here we reveal genera with high centrality but low research attention as prime candidates for future study. Both pollinator management and sociality have an impact on research effort. Excluding *Apis* and *Bombus* (the most traditionally researched genera), managed bee genera still receive twice as many papers as wild genera, with the managed share rising over time. Our study reveals and quantifies persistent global research biases and highlights the need for monitoring, risk assessment, and policies that target neglected yet structurally central genera in recorded visitation networks.

Supplementary Material

Supplementary Results: Geography and Income Pattern of Managed-Bee Emphasis

Across 169 countries and 3,153 country–years (1975–2023), we modelled the probability that a country–year paper focused on managed bees using a binomial GLMM with country random intercepts and fixed effects for income group, region, and year (Supplementary Fig. 1). Relative to high-income countries, lower-middle-income settings show lower odds of a managed-bee focus (OR = 0.62, 95% CI 0.39–0.98; $p = 0.042$), while low-income (0.73, 0.36–1.49; $p = 0.389$) and upper-middle-income (0.92, 0.64–1.33; $p = 0.668$) are not distinguishable from high income. By region (baseline East Asia & Pacific), estimated differences are imprecise: Latin America & Caribbean is lower (0.62, 0.37–1.03; $p = 0.067$) and Europe & Central Asia is higher (1.45, 0.92–2.30; $p = 0.107$), but neither reaches $\alpha = 0.05$ in the primary specification; other regions are similarly not distinguishable at $\alpha = 0.05$. A positive year effect indicates that the worldwide share of managed-bee papers has risen over time (per-SD OR = 1.17, 1.15–1.20; $p < 0.001$). These adjusted contrasts are shown in Supplementary Fig. 1B (income) and Supplementary Fig. 1C (region). See supplemental dataset 5 for sample sizes.

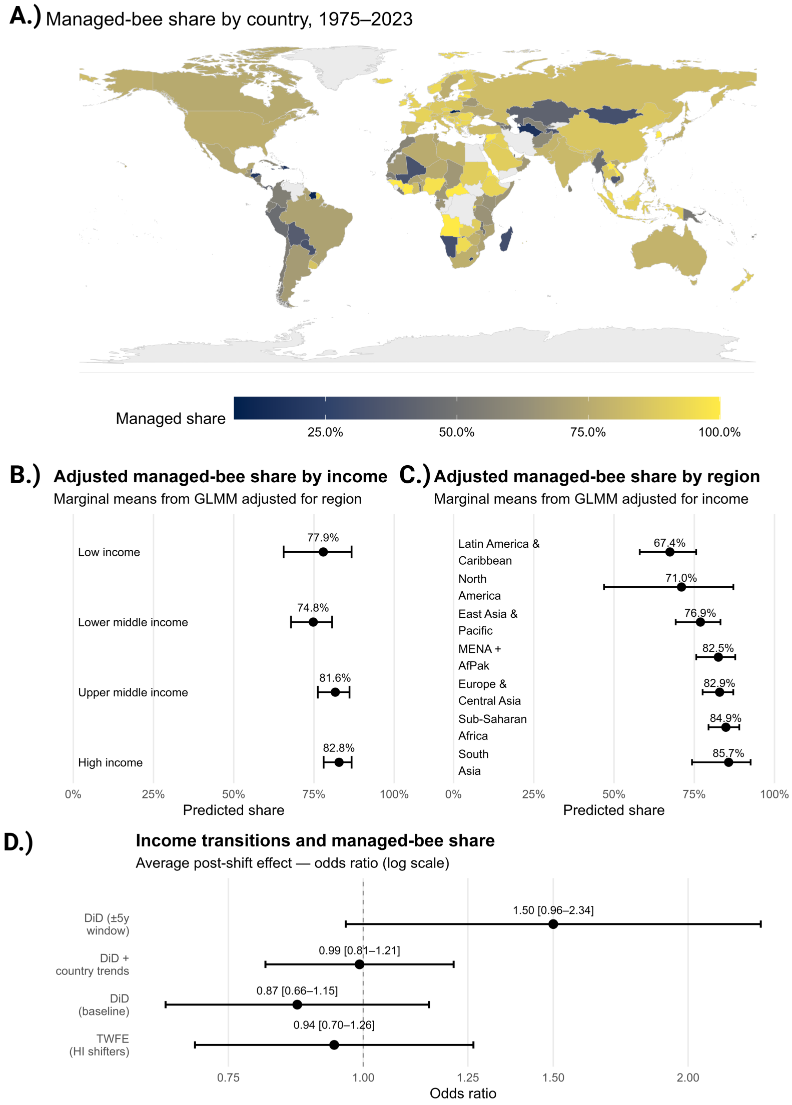

Figure 1: **Supplementary Data Figure 1.) Geographic patterning of the managed-bee literature and adjusted group contrasts.** A.) Country-level share of papers that focus on managed bees, aggregated over 1975–2023. Colours show the fraction of a country's bee-genus papers that are on managed taxa (dark yellow = higher share; dark blue = lower share; light grey = no data). B.) Predicted managed-bee share by World Bank income group from a binomial GLMM with country random intercepts and fixed effects for income group, region, and year (fit on country-year data; n = 3,153 country–years; 169 countries). Points are marginal means on the probability scale (year at the sample mean); horizontal bars are 95% CIs; values are printed above points. C.) As in panel B but by world region (labels shortened for readability: 'MENA + AfPak' denotes Middle East, North Africa, Afghanistan & Pakistan). D.) Income-transition effects on managed-bee share across specifications (odds ratios with 95% CIs, log scale): TWFE (two-way fixed effects model) among high-income 'shifters' (0.79, 0.54–1.15), TWFE with country trends (0.78, 0.57–1.09), DiD (Difference-in-Differences) baseline (1.13, 0.92–1.39), DiD with country trends (1.14, 0.97–1.33), and DiD in a ±5-year window (1.47, 1.01–2.13).

At the genus level, managed taxa receive more papers per country–year output. In a negative-binomial model of genus-level article counts (offset by country–year output; random intercepts for genera and countries), managed genera receive about 2.09× the counts of wild genera (IRR = 2.09, 1.63–2.68; $p < 0.001$). The sociality effect is weaker and not distinguishable from 1 in this specification (IRR = 1.18, 0.94–1.48; $p = 0.163$). After covariates

and year are controlled, low-, lower-middle-, and upper-middle-income settings show higher per-genus counts than high-income (IRRs = 1.74, 1.71, 1.36; all $p \leq 0.003$). Regionally, Latin America & Caribbean is above the baseline (1.42, 1.08–1.88), while North America (0.40, 0.22–0.73) and South Asia (0.64, 0.41–0.99) are below; other regions are not distinguishable in this model.

For income transitions we restricted the analysis to countries that changed income tier and estimated within-country effects on managed share using two-way fixed effects and difference-in-differences. We implemented an event-study using the Sun–Abraham estimator to visualise dynamic effects around the first upward shift and included a version with linear country trends. We performed leave-one-country-out summaries and placebo checks, and we documented a narrow five-year window analysis as a robustness check(1, 2, 3). Event-study and TWFE estimators were implemented with `fixest::sunab`(4). Welch ANOVA post-hoc contrasts used `rstatix` for Games–Howell (5)

Income transitions upwards show specification-dependent changes towards managed bees. Using only countries that change status, TWFE estimates among high-income 'shifters' are near-null (OR = 0.79, 0.54–1.15; with country trends 0.78, 0.57–1.09). DiD on the first upward income shift is small and uncertain (1.13, 0.92–1.39; with country trends 1.14, 0.97–1.33). A restricted ±5-year window yields a larger estimate (1.47, 1.01–2.13), indicating that inference on transitions is sensitive to specification and window choice.

Patterns extend to sociality and nesting compositions but remain imprecise. TWFE on the share of social bees suggests lower odds in high-income 'shifters' but with uncertainty (OR = 0.82, 0.58–1.17; with country trends 0.76, 0.58–1.01). For nesting type, DiD around the first upward shift indicates modest changes: large-colony cavity nesting increases (OR = 1.17, 0.96–1.42), while wood-excavating decreases (0.81, 0.62–1.05).

Supplementary Results: VIF Analysis

To evaluate multicollinearity among fixed effects in the negative-binomial count models (full dataset; and a sensitivity model excluding *Apis* and *Bombus*), we calculated variance inflation factors (VIFs) for the full fixed-effect specifications, including the Managed × Year interaction.

Across both models, VIFs were low for the focal predictors and the interaction term (full dataset: Managed = 1.88; Year = 1.15; log(species richness) = 1.12; Managed × Year = 1.14; excluding *Apis*/*Bombus*: Managed = 2.01; Year = 1.15; log(species richness) = 1.13; Managed × Year = 1.18), indicating limited collinearity among the main inferential terms.

In contrast, Sociality and Nesting type exhibited elevated VIFs (full dataset: Sociality = 8.76; Nesting type = 9.43; excluding *Apis*/*Bombus*: Sociality = 12.80; Nesting type = 15.54). This pattern is expected given strong structural association between these life-history traits and management status (and substantial imbalance/sparsity across levels; e.g., very few wood-excavating and cavity-nesting genera). We therefore interpret coefficients for Sociality

and Nesting type with caution, but note that multicollinearity does not appear to meaningfully affect estimation of the primary effects of interest (Managed, Year, species richness) or the Managed × Year interaction, which all showed consistently low VIFs.

Supplementary Tables

Table 1: Detailed Database Search Strategy. Keywords used in the WoS and Scopus searches by topic. This table provides the detailed search queries and keywords used to assemble the publication corpus from the Web of Science (WoS) and Scopus databases.

Database	Search Details	Results
Scopus	**Search Period:** 1950–2024<br>**Topic Query String:**<br>TITLE-ABS-KEY (bee OR bees OR beekeeping OR Apoidea OR "honey bee" OR "bumble bee" OR "solitary bee" OR "stingless bee" OR Apis OR Bombus OR Apidae OR Halictidae OR Megachilidae OR Andrenidae OR Colletidae OR Osmia OR Xylocopa OR Megachile OR Lasioglossum)<br>**Filters & Refinements:**<br>• **Publication Years:** 1950–2024 (Query: PUBYEAR > 1949 AND PUBYEAR < 2025)<br>• **Subject Areas:**<br>– AGRI (Agricultural and Biological Sciences)<br>– BIOC (Biochemistry, Genetics and Molecular Biology)<br>– DECI (Decision Sciences)<br>– ECON (Economics, Econometrics and Finance)<br>– ENVI (Environmental Science)<br>– IMMU (Immunology and Microbiology)<br>– MULT (Multidisciplinary)<br>– NEUR (Neuroscience)<br>– SOCI (Social Sciences)<br>– VETE (Veterinary)<br>– ARTS (Arts and Humanities)<br>**Processing Notes:**<br>• Results were downloaded in batches by date range (1950–2007, 2007–2015, 2015–2019, 2019–2023, 2021–2024).<br>• Batches were concatenated into a single file and initial duplicates were removed.	65,431

Table 1: Detailed Database Search Strategy (Continued)

Database	Search Details	Results
Web of Science	**Search Period:** 1950–2024<br>**Collections Searched:**<br>• Web of Science Core Collection, CABI: CAB Abstracts®, BIOSIS Citation Index, MEDLINE®, SciELO Citation Index, KCI-Korean Journal Database<br>**Topic Query String:**<br>`TS=(bee OR bees OR beekeeping OR Apoidea OR "honey bee" OR "bumble bee" OR "solitary bee" OR "stingless bee" OR Apis OR Bombus OR Apidae OR Halictidae OR Megachilidae OR Andrenidae OR Colletidae OR Osmia OR Xylocopa OR Megachile OR Lasioglossum)`<br>**Filters & Refinements:**<br>• **Publication Years:** 1950–2024 (specified as a list from 2023 down to 1950 in the query)<br>• **Database Exclusions:** Preprint Citation Index<br>• **Document Types:** Article, Review Article, Dissertation Thesis<br>• **Language:** English<br>• **Research Areas:**<br>– Agriculture<br>– Allergy<br>– Anatomy Morphology<br>– Anthropology<br>– Behavioral Sciences<br>– Biochemistry Molecular Biology<br>– Biodiversity Conservation<br>– Biophysics<br>– Biotechnology Applied Microbiology<br>– Cardiovascular System Cardiology<br>– Cell Biology<br>– Entomology<br>– Environmental Sciences Ecology<br>– Evolutionary Biology<br>– Food Science Technology<br>– Forestry<br>– Gastroenterology Hepatology<br>– Genetics Heredity<br>– History<br>– Immunology<br>– Infectious Diseases<br>– Life Sciences Biomedicine Other Topics<br>– Marine Freshwater Biology<br>– Meteorology Atmospheric Sciences<br>– Microbiology<br>– Microscopy<br>– Mycology<br>– Nutrition Dietetics<br>– Parasitology<br>– Pathology<br>– Physiology<br>– Plant Sciences<br>– Public Environmental Occupational Health<br>– Reproductive Biology<br>– Science Technology Other Topics<br>– Social Issues<br>– Social Sciences Other Topics<br>– Toxicology<br>– Urban Studies<br>– Veterinary Sciences<br>– Virology<br>– Zoology	92,889

**Bee genus**	**Pollination service value**	**Honey & wax production value**	**Key sources**
Apis	Global: \$200–300 billion (Gallai *et al.* 2009). U.S.: \$15 billion yr^{-1} (USDA); \$11.7 billion (Calderone 2012). U.K.: ∼34% (£150–200 million).	Global: \$8–9 billion honey; \$0.6 billion beeswax (FAO). U.S.: \$300–350 million yr^{-1} honey. U.K.: <£20 million yr^{-1}.	Gallai *et al.* (6); Calderone (7); USDA (8); DEFRA (9)
Bombus	U.S.: Part of \$9 billion wild-pollinator contribution (White House 2015); \$690 million for greenhouse tomatoes. U.K.: ∼66% of non-honey-bee pollination (£400 million).	—	White House (10); DEFRA (9); OSU (11)
Megachile	U.S.: \$5–7 billion yr^{-1} in alfalfa hay.	—	Calderone (7)
Osmia	Orchard pollinators; can double cherry yield. Japan: *O. cornifrons* widely used.	—	USDA ARS (12); Bosch & Kemp (13); McKinney & Park (14)

Table 2: Estimated Annual Economic Value of Selected Bee Species. This table presents a comprehensive summary of the estimated annual economic contributions of key bee species based on their roles in crop pollination and hive product production. The values, provided in US dollars for global and U.S. estimates and in British pounds for U.K. data, are derived from a variety of peer-reviewed studies and government reports. Each row corresponds to a specific bee species or group and includes an estimate of its contribution to pollination services across different regions, and the associated economic value from honey and beeswax production (where applicable). Notably, the western honey bee (*Apis mellifera*) is recognised for its dominant global pollination impact, while bumblebees (*Bombus* spp.) are critical in both wild and greenhouse settings despite limited honey production. The alfalfa leafcutter bee (*Megachile rotundata*) plays a pivotal role in alfalfa seed production, and mason bees (*Osmia* spp.) are important orchard pollinators.

Supplementary Table 3: Tractability — Bee Species Reared in Laboratory Conditions

Genus	Species	Frequency	Purpose	Rearing Success / Notes
Apis	*mellifera*	Very common	Research (behaviour, genetics, toxicology), pollination, honey/venom production	High success; globally standardised protocols, in-vitro larval rearing routinely exceeds 95% survival (15, 16, 17, 18)
Apis	*cerana*	Common (Asia)	Pollination, honey production, comparative research	Moderate success; proven hive and queen-rearing methods but colonies may abscond if stressed (19, 20)
Bombus	*terrestris*	Very common	Greenhouse pollination (tomatoes, peppers), research (behaviour, ecotoxicology)	High success; first bumblebee commercialised worldwide, millions of colonies reared annually (21, 22, 23, 24, 25)
Bombus	*impatiens*	Very common (N. America)	Greenhouse pollination, research (behaviour, pesticide studies)	High success; main commercial bumblebee in North America—standardised rearing, but queen founding remains the bottleneck (26, 27, 25)
Bombus	*occidentalis*	Historically common, now occasional	Greenhouse pollination, conservation rearing	Once widely reared; severely impacted by pathogens; limited conservation breeding continues (25, 28)

Continued on next page

**Genus**	**Species**	**Frequency**	**Purpose**	**Rearing Success / Notes**
Bombus	*ignitus*	Common (E. Asia)	Pollination, research	Commercially reared in Asia; protocols parallel those for *B. terrestris* (29)
Bombus	*lucorum* (complex)	Occasional	Pollination trials, research	Moderate success; can be reared like *B. terrestris* but little commercial demand (25, 30, 31)
Bombus	*atratus*	Occasional (S. America)	Pollination, tropical-bumblebee studies	Developing methods; multiyear colony cycles complicate rearing (32, 33)
Bombus	*ephippiatus*	Rare (experimental)	Greenhouse tomato pollination, local research	Limited success in laboratory colony initiation; feasible but not routine (34, 35)
Bombus	*huntii*	Rare	Research, small-scale pollination	Regionally tested in W. North America; rearing protocols exist but not commercialised (36, 37)
Nomia	*melanderi*	Regionally common (field-managed)	Alfalfa pollination, research	High success in specialised "bee beds" with saline soil; seldom reared indoors (38, 39)
Lasioglossum	*zephyrum*	Occasional (research)	Behavioural and social-structure studies	Moderate success in soil-sandwich nest boxes; classic sweat-bee model (39)

Continued on next page

Genus	Species	Frequency	Purpose	Rearing Success / Notes
Osmia	*lignaria*	Common (managed)	Orchard pollination (almonds, apples), research	High success in artificial nesting blocks/tubes; widely used in N. America (40)
Osmia	*bicornis* (syn. *rufa*)	Common (Europe)	Orchard/berry pollination, research	High success; readily nests in cardboard or bamboo tubes (41)
Osmia	*cornifrons*	Common (Japan; introduced USA)	Orchard pollination (apples), research	High success; mass-produced in Japan and trialled in the USA (42, 43)
Osmia	*ribifloris*	Rare (experimental)	Blueberry pollination, toxicology research	Moderate success with in-vitro larval rearing; *Vaccinium* specialist hinders scaling (44)
Megachile	*rotundata*	Very common (global)	Alfalfa/clover pollination, research	Highly successful commercial rearing; billions released annually (13, 45)
Xylocopa	*violacea*	Rare (experimental)	Greenhouse pollination research	Challenging to complete the life-cycle indoors; partial success in wooden blocks (46)
Melipona	*scutellaris*	Rare (research)	Toxicology tests, conservation	In-vitro larval protocols recently developed; moderate success but difficult to scale (47, 48)
Scaptotrigona	*postica*	Rare (experimental)	Larval toxicology, breeding research	Limited success with artificial brood; methods still under refinement (49)

Continued on next page

Genus	Species	Frequency	Purpose	Rearing Success / Notes
Tetragonisca	*angustula*	Rare (experimental)	Pesticide-impact studies, meliponiculture	In-vitro larval rearing shows partial success; natural brood care complex to mimic (50)

Table 3: Bee species reared under laboratory or controlled conditions, organised by genus and species. For each species we report (i) the frequency with which the species is reared in laboratory settings (ranging from "Very common" to "Rare"), (ii) the primary purposes for which the species is reared—including applications in research (e.g., behaviour, genetics, toxicology), pollination services in controlled agricultural environments, production (e.g., honey, venom extraction), and conservation—and (iii) qualitative notes regarding the success and challenges associated with rearing each species. Notable examples include *Apis mellifera*, which is globally reared with in-vitro protocols achieving greater than 95% larval survival, and various *Bombus* species that have been commercially reared for greenhouse pollination. Lesser-known species, such as *Scaptotrigona postica* and *Tetragonisca angustula*, are included to highlight emerging laboratory techniques and conservation breeding efforts.

Supplementary Table 4: Leave-One-Out Analysis of Regions

Excluded region	n networks	n genera	Spearman's ρ	p-value
Latin America & Caribbean	65	66	0.012	0.924
North America	81	87	0.225	0.036
Europe & Central Asia	76	82	0.190	0.088
East Asia & Pacific	78	97	0.146	0.155
Sub-Saharan Africa	60	100	0.182	0.070

Table 4: Leave-one-out analysis of world regions for the genus-level centrality–effort association. Each row reports results from re-running the genus-level analysis after excluding all plant–pollinator (visitation) networks from one world region at a time (region assignments from Web of Life metadata). For each reduced dataset, genus centrality was recomputed from the remaining networks using the same within-network standardisation and cross-network aggregation as in the main analysis, and the association with research effort was quantified using Spearman's rank correlation (ρ; two-sided p-values). Correlations remain weak across all exclusions ($\rho = 0.012$–0.225), with only the North America exclusion reaching $\alpha = 0.05$ ($p = 0.036$); all other exclusions $p \geq 0.070$. This indicates that the centrality–effort pattern is not attributable to any single region.

Supplementary Figures

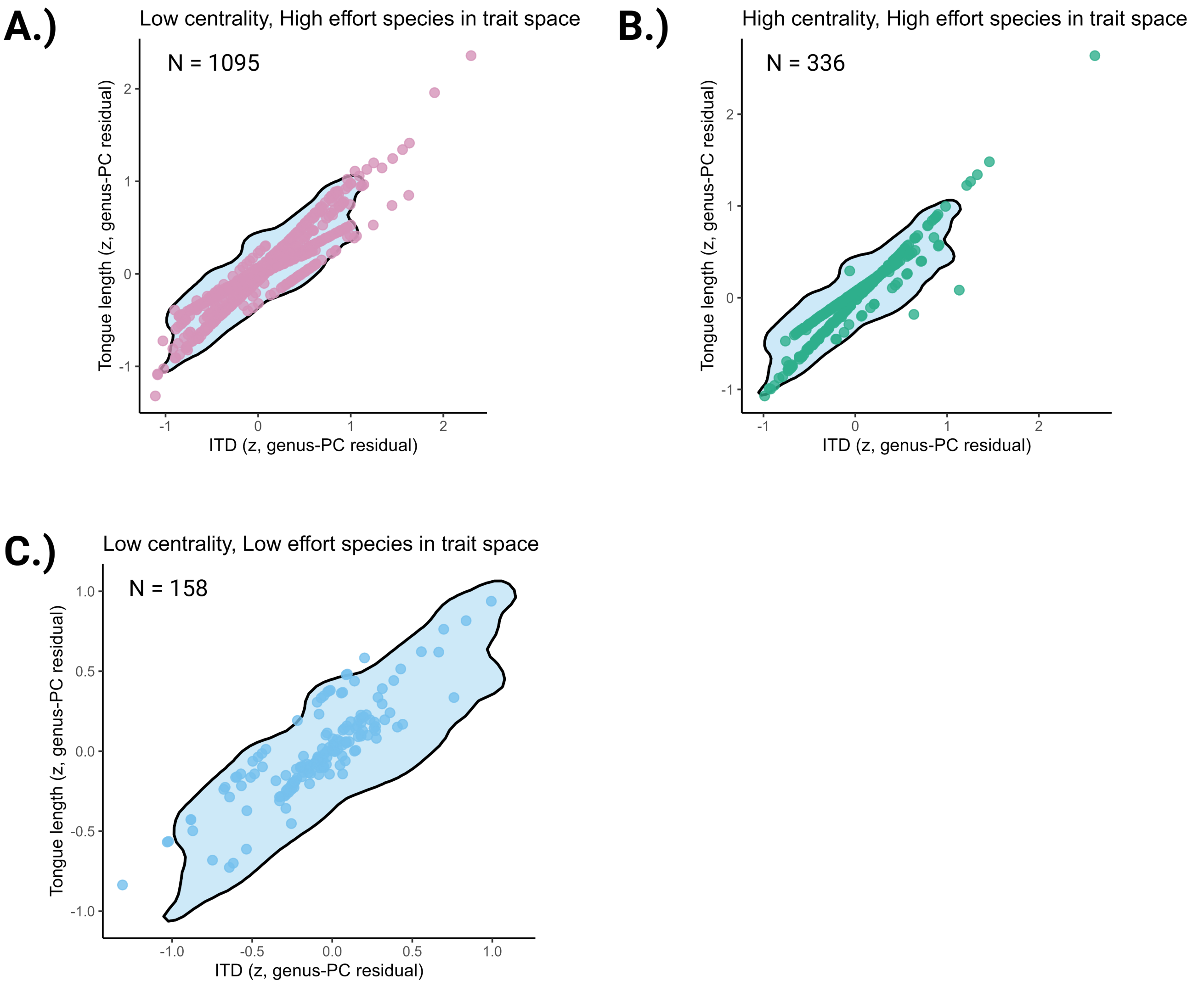

Figure 2: **Supplementary Data Figure 2.** Trait space of species: positions of species traits by classification over the background envelope of all other species (teal, kernel-density isopleth). Axes are genus-PC-residualised z-scores for intertegular distance (ITD) and tongue length.

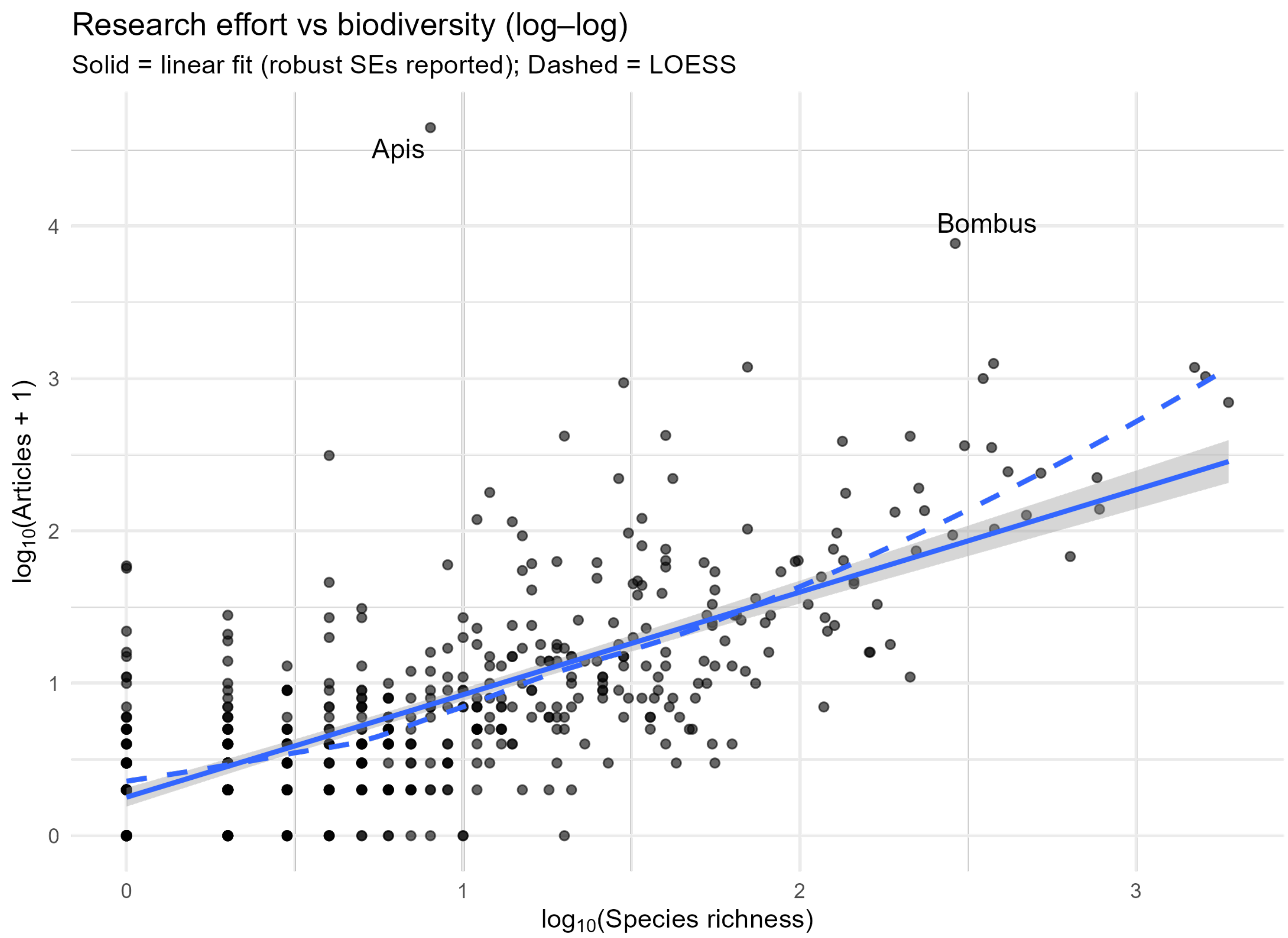

Figure 3: **Supplementary Data Figure 3: Alternative fit for richness–effort scaling.** Comparison of the log–log linear fit (solid) and a LOESS smoother (dashed) shows similar curvature, indicating the sub-linear scaling is not an artifact of model form. Sensitivity analyses removing high-influence genera and excluding *Apis*/*Bombus* yield slopes of 0.583 and 0.660 (95% CIs shown), respectively, consistent with sub-linear scaling.

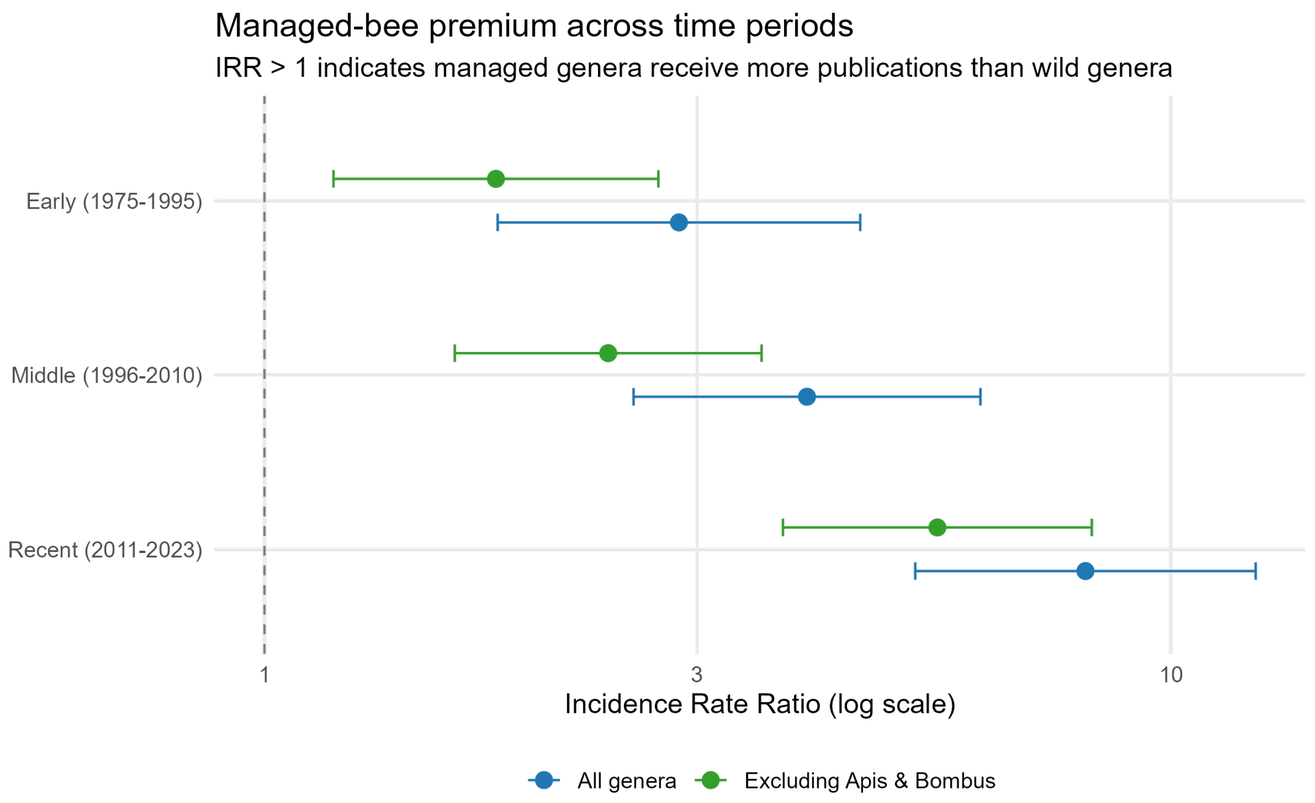

Figure 4: **Supplementary Data Figure 4: Managed-bee premium strengthens over time.** Points show incidence rate ratios (IRR) for publication counts of managed genera relative to wild genera, estimated from negative-binomial mixed-effects models fit separately for three time windows: early (1975–1995), middle (1996–2010), and recent (2011–2023). Horizontal bars indicate 95% confidence intervals and the vertical dashed line marks IRR = 1 (no managed advantage). Blue points use all genera; green points repeat the analysis excluding *Apis* and *Bombus*. The managed premium increases across periods in both datasets (all genera: IRR 2.87, 3.97, 8.05; excluding *Apis* and *Bombus*: IRR 1.80, 2.39, 5.52; x-axis on a log scale).

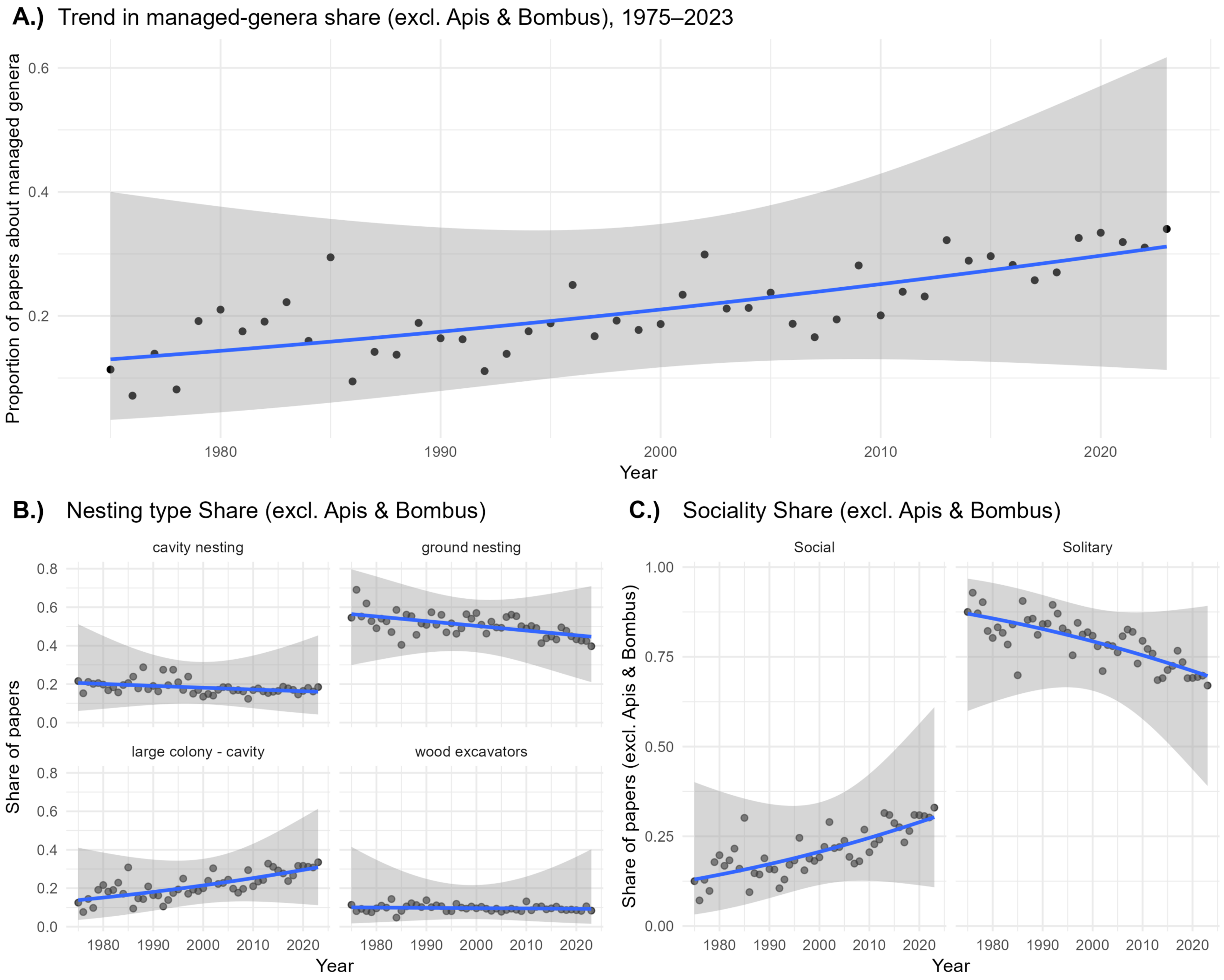

Figure 5: **Supplementary Data Figure 5:** Drivers and temporal shifts in global bee research effort excluding the dominant genera *Apis* and *Bombus*. (A) Predictors of research attention for the 'no-AB' dataset. Points represent Incident Rate Ratios (IRR) from the count model, showing that species richness and wood-excavation are the primary positive drivers of effort when the most-studied taxa are excluded. (B) Growth in the research share of managed lineages from 1980 to the present. The sharp increase ($\beta = +0.0259\ yr^{-1}$) indicates a systemic shift in the field toward managed taxa. (C) Comparison of temporal shifts in research share between social and solitary taxa. The gain in social taxa share mirrors the decline in solitary taxa, confirming a broad reweighting of focus toward social lineages. Error bars and shaded areas represent 95% confidence intervals.

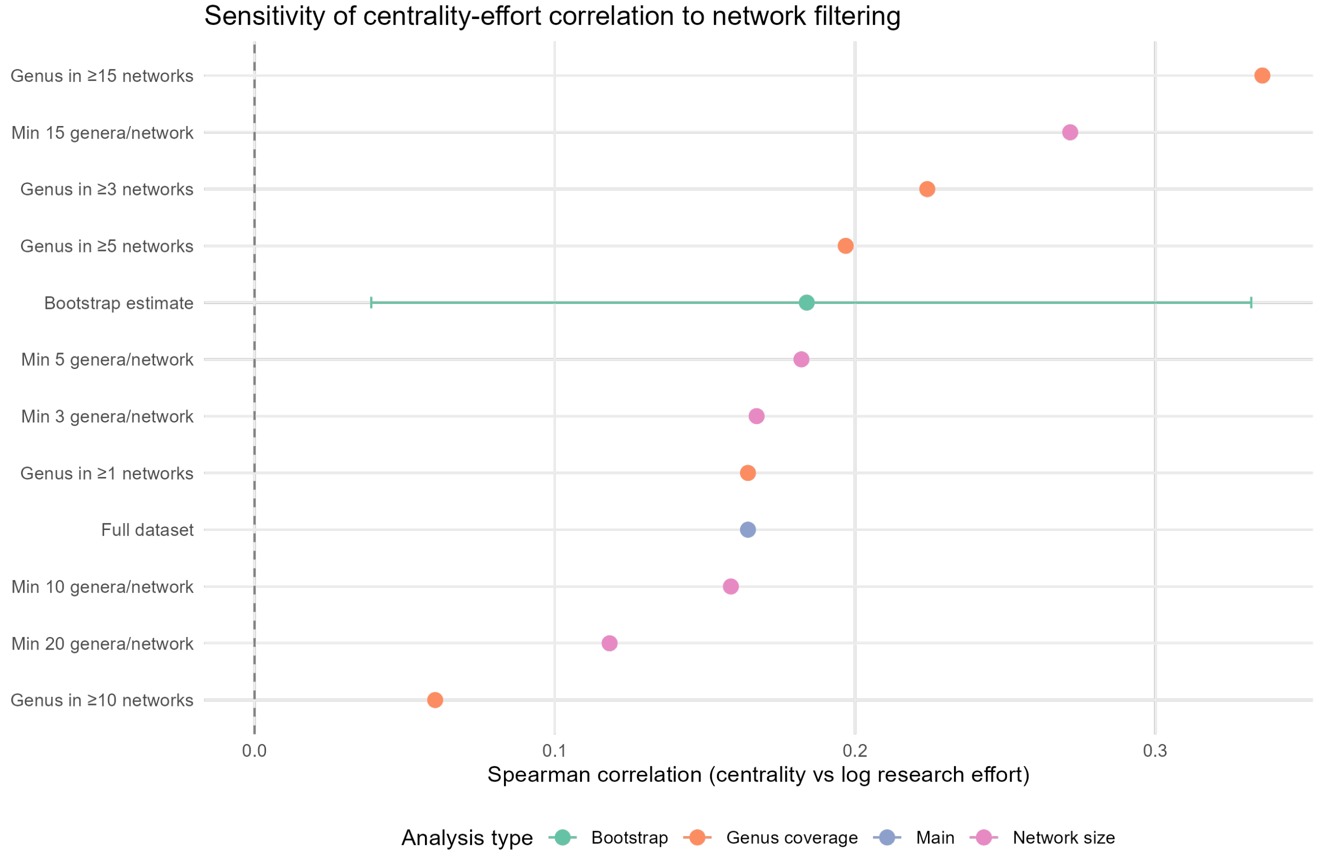

Figure 6: **Supplementary Data Figure 6: Network heterogeneity sensitivity analysis for the genus-level centrality–effort association.** Points show Spearman's rank correlation (ρ) between genus mean ecological centrality and research effort across Web of Life plant–pollinator interaction networks, under alternative inclusion and filtering rules. Centrality was quantified as pollinator species strength (bipartite species-level) within each network, z-standardised within network to remove scale differences among networks and then averaged across all networks in which a genus occurred to obtain a genus-level mean (z-strength). Research effort was measured as $\log(articles + 1)$ using machine-learning-assisted genus article counts aggregated to total articles per genus. The full dataset includes 89 networks (bee genera per network: median 5, range 1–40; total interactions per network: median 243, range 65–28,224) and 101 genera with both centrality and effort. Sensitivity conditions include: genus coverage thresholds (restricting to genera observed in ≥ 1, ≥ 3, ≥ 5, ≥ 10, and ≥ 15 networks; $\rho = 0.164, 0.224, 0.197, 0.060, 0.336$, respectively; $n = 100, 42, 30, 20, 12$), and network size filtering (restricting to networks containing at least 3, 5, 10, 15, or 20 bee genera; $\rho = 0.261, 0.266, 0.243, 0.396, 0.180$; networks retained $= 82, 50, 30, 9, 4$). A network bootstrap (1,000 resamples of networks with replacement; green point) quantifies uncertainty in the correlation to network composition (bootstrap mean $\rho = 0.184$; 95% CI $= 0.039$–0.332; horizontal error bar). The vertical dashed line indicates $\rho = 0$ (no association). Overall, the correlation remains positive across most filtering choices, indicating that the observed weak centrality–effort coupling is not driven solely by differences in network size, sparsely represented genera, or any single sampling rule.

Supplementary Data

Code and derived, aggregated datasets are available at Zenodo: Nesbit, M. (2026). Mapping global bee research with traits and plant-pollinator visitation networks - code and data. Zenodo. DOI: 10.5281/zenodo.17505759. Licensed bibliographic corpora (e.g., Web of Science/SCOPUS) cannot be redistributed and are available from the original providers under their terms.

Dataset 1–4: Species categories for effort and traits.

Dataset 5: Country sample sizes.

References

[1] Brantly Callaway and Pedro H. C. Sant'Anna. Difference-in-Differences with multiple time periods. *Journal of Econometrics*, 225(2):200–230, 2021. doi: 10.1016/j.jeconom.2020.12.001.

[2] Clément de Chaisemartin and Xavier d'Haultfoeuille. Two-Way Fixed Effects Estimators with Heterogeneous Treatment Effects. *American Economic Review*, 110(9):2964–2996, 2020. doi: 10.1257/aer.20181169.

[3] Liyang Sun and Sarah Abraham. Estimating dynamic treatment effects in event studies with heterogeneous treatment effects. *Journal of Econometrics*, 225(2):175–199, 2021. doi: 10.1016/j.jeconom.2020.09.006.

[4] Laurent Berge. *fixest: Fast Fixed-Effects Estimations.* 2024. URL `https://CRAN.R-project.org/package=fixest`.

[5] Alboukadel Kassambara. *rstatix: Pipe-Friendly Framework for Basic Statistical Tests.* 2024. URL `https://CRAN.R-project.org/package=rstatix`.

[6] Nicola Gallai, Jean-Michel Salles, Josef Settele, and Bernard E. Vaissière. Economic valuation of the vulnerability of world agriculture confronted with pollinator decline. *Ecological Economics*, 68(3):810–821, January 2009. ISSN 0921-8009. doi: 10.1016/j.ecolecon.2008.06.014. URL `http://dx.doi.org/10.1016/j.ecolecon.2008.06.014`.

[7] Nicholas W Calderone. Insect Pollinated Crops , Insect Pollinators and US Agriculture : Trend Analysis of Aggregate Data for the Period 1992 – 2009. 7(5):24–28, 2012. doi: 10.1371/journal.pone.0037235.

[8] USDA National Agricultural Statistics Service. Honey Report: 2023 Summary. Technical report, U.S. Department of Agriculture, Washington, DC, 2024.

[9] Adam J. Vanbergen, Matt S. Heard, Tom D. Breeze, Simon G. Potts, and Nick Hanley. Status and Value of Pollinators and Pollination Services. Report, Department for Environment, Food & Rural Affairs, London, UK, March 2014.

[10] Pollinator Health Task Force. National Strategy to Promote the Health of Honey Bees and Other Pollinators. Technical report, Executive Office of the President of the United States, Washington, DC, May 2015.

[11] Emily Runnion. Bumble Bee Pollination in Tomato Greenhouses. Fact Sheet ENT-0092, Ohio State University Extension, Columbus, OH, 2023.

[12] USDA Agricultural Research Service. Blue Orchard Bee (*Osmia lignaria*): Commercial Pollinator for Orchard Crops. Fact Sheet, Pollinating Insect–Biology, Management and Systematics Research Unit, Logan, UT, 2021.

[13] WP Kemp and Jordi Bosch. Development and emergence of the alfalfa pollinator *Megachile rotundata* (Hymenoptera: Megachilidae). *Annals of the Entomological Society of America*, 93(4):904–911, 2000.

[14] Matthew I. McKinney and Yong-Lak Park. Nesting activity and behavior of *Osmia cornifrons* (Hymenoptera: Megachilidae) elucidated using videography. *Psyche: A Journal of Entomology*, 2012(1):814097, 2012. ISSN 1687-7438. doi: 10.1155/2012/814097. URL https://onlinelibrary.wiley.com/doi/abs/10.1155/2012/814097. _eprint: https://onlinelibrary.wiley.com/doi/pdf/10.1155/2012/814097.

[15] Marina Carla Bezerra da Silva, Madison Gail Kindopp, Midhun Sebastian Jose, Oleksii Obshta, Thanuri Lakna Kumari Edirithilake, Emilio Enrique Tellarini Prieto, Muhammad Fahim Raza, Marcelo Polizel Camilli, Jenna Thebeau, Fatima Masood, Ivanna Kozii, Igor Moshynskyy, Elemir Simko, and Sarah C. Wood. From larva to adult: In vitro rearing protocol for honey bee (*Apis mellifera*) drones. *PLOS ONE*, 20(2):e0314859, February 2025. ISSN 1932-6203. doi: 10.1371/journal.pone.0314859. URL https://www.ncbi.nlm.nih.gov/pmc/articles/PMC11824949/.

[16] Karl Crailsheim, Robert Brodschneider, Pierrick Aupinel, Dieter Behrens, Elke Genersch, Jutta Vollmann, and Ulrike Riessberger-Gallé. Standard methods for artificial rearing of Apis mellifera larvae. *Journal of Apicultural Research*, 52(1):1–16, January 2013. ISSN 0021-8839. doi: 10.3896/IBRA.1.52.1.05. URL https://doi.org/10.3896/IBRA.1.52.1.05. _eprint: https://doi.org/10.3896/IBRA.1.52.1.05.

[17] Daniel R Schmehl, Hudson V V Tomé, Ashley N Mortensen, Gustavo Ferreira Martins, and James D Ellis. Protocol for the in vitro rearing of honey bee (*Apis mellifera* L.) workers. *Journal of Apicultural Research*, 55(2):113–129, March 2016. ISSN 0021-8839. doi: 10.1080/00218839.2016.1203530. URL https://www.tandfonline.com/doi/full/10.1080/00218839.2016.1203530.

[18] Ralph Büchler, Andonov , Sreten, Bienefeld , Kaspar, Costa , Cecilia, Hatjina , Fani, Kezic , Nikola, Kryger , Per, Spivak , Marla, Uzunov , Aleksandar, , and Jerzy Wilde. Standard methods for rearing and selection of *Apis mellifera* queens. *Journal of Apicultural Research*, 52(1):1–30, January 2013. ISSN 0021-8839. doi: 10.3896/IBRA.1.52.1.07. URL https://doi.org/10.3896/IBRA.1.52.1.07. _eprint: https://doi.org/10.3896/IBRA.1.52.1.07.

[19] Dharam Pal Abrol, R. M. Bhagat, and Devinder Sharma. Mass Rearing of *Apis cerana* F. Queen. *Journal of Asia-Pacific Entomology*, 8(3):309–317, September 2005. ISSN 1226-8615. doi: 10.1016/S1226-8615(08)60251-4. URL https://www.sciencedirect.com/science/article/pii/S1226861508602514.

[20] Eva Crane. *Apis* species of tropical Asia as pollinators, and some rearing methods for them. In *VI International Symposium on Pollination 288*, pages 29–48, 1990.

[21] Peter-Frank Röseler. A technique for year-round rearing of *Bombus terrestris* (Apidae, Bombini) colonies in captivity. *Apidologie*, 16(2):165–170, 1985.

[22] Dave Goulson, Pippa Rayner, Bob Dawson, and Ben Darvill. Translating research into action; bumblebee conservation as a case study. *Journal of Applied Ecology*, 48(1):3–8, December 2010. ISSN 0021-8901. doi: 10.1111/j.1365-2664.2010.01929.x. URL http://dx.doi.org/10.1111/j.1365-2664.2010.01929.x.

[23] Dave Goulson, Elizabeth Nicholls, Cristina Botías, and Ellen L. Rotheray. Bee declines driven by combined stress from parasites, pesticides, and lack of flowers. *Science*, 347(6229), March 2015. ISSN 0036-8075. doi: 10.1126/science.1255957. URL http://dx.doi.org/10.1126/science.1255957.

[24] Penelope R Whitehorn, Matthew C Tinsley, Mark J F Brown, and Dave Goulson. Investigating the impact of deploying commercial Bombus terrestris for crop pollination on pathogen dynamics in wild bumble bees. *Journal of Apicultural Research*, 52(3):149–157, 2013. ISSN 0021-8839.

[25] H. H. W. Velthuis and Adriaan van Doorn. A century of advances in bumblebee domestication and the economic and environmental aspects of its commercialization for pollination. *Apidologie*, 37(4):421–451, 2006. doi: 10.1051/apido:2006019.

[26] Erin Treanore, Katherine Barie, Nathan Derstine, Kaitlin Gadebusch, Margarita Orlova, Monique Porter, Frederick Purnell, and Etya Amsalem. Optimizing laboratory rearing of a key pollinator, *Bombus impatiens*. *Insects*, 12(8):673, August 2021. ISSN 2075-4450. doi: 10.3390/insects12080673. URL https://www.mdpi.com/2075-4450/12/8/673. Number: 8.

[27] J. Cnaani, R. Schmid-Hempel, and J.O. Schmidt. Colony development, larval development and worker reproduction in *Bombus impatiens Cresson*. *Insectes Sociaux*, 49(2):164–170, May 2002. ISSN 1420-9098. doi: 10.1007/s00040-002-8297-8. URL https://doi.org/10.1007/s00040-002-8297-8.

[28] Sujaya Rao and William P. Stephen. *Bombus (Bombus) occidentalis* (Hymenoptera: Apiformes): In decline or recovery. *The Pan-Pacific Entomologist*, 83(4):360–362, December 2007. ISSN 0031-0603, 2162-0237. doi: 10.3956/2007-10.1. URL https://bioone.org/journals/the-pan-pacific-entomologist/volume-83/issue-4/2007-10.1/Bombus-Bombus-occidentalis-Hymenoptera--Apiformes--In-decline-or/10.3956/2007-10.1.full.

[29] Hyung Joo Yoon, Kyeong Yong Lee, Mi Ae Kim, Sang Mi Han, and In Gyun Park. Breeding of the Korean Native Bumblebee, *Bombus ignitus*. *Journal of Apiculture*, 27(3):179–186, October 2012. ISSN 1225-0252. URL https://www.dbpia.co.kr/Journal/articleDetail?nodeId=NODE02049820.

[30] Alena Bučánková and Vladimír Ptáček. Experiences in rearing of *Bombus lucorum* L.(Hymenoptera: Apoidea) in captivity. *Úroda*, 12:621–624, 2010.

[31] Jilian Li, Wu , Jie, Cai , Wanzhi, Peng , Wenjun, An , Jiandong, , and Jiaxing Huang. Comparison of the colony development of two native bumblebee species *Bombus ignitus* and *Bombus lucorum* as candidates for commercial pollination in China. *Journal of Apicultural Research*, 47(1):22–26, January 2008. ISSN 0021-8839. doi: 10.1080/00218839.2008.11101419. URL `https://doi.org/10.1080/00218839.2008.11101419`. _eprint: https://doi.org/10.1080/00218839.2008.11101419.

[32] Maria Teresa Almanza Fandiño. *Management of Bombus atratus bumblebees to pollinate lulo (Solanum quitoense L), a native fruit from the Andes of Colombia*, volume 50. Cuvillier Verlag, 2007.

[33] Sheena Salvarrey. Artificial Breeding of Native Bumblebees *Bombus atratus* and *Bombus bellicosus* (Hymenoptera, Apidae), 2013.

[34] Carlos Hernan Vergara and Paula Fonseca-Buendía. Pollination of greenhouse tomatoes by the Mexican bumblebee *Bombus ephippiatus* (Hymenoptera: Apidae). *Journal of Pollination Ecology*, 7, April 2012. ISSN 1920-7603. doi: 10.26786/1920-7603(2012)1. URL `https://pollinationecology.org/index.php/jpe/article/view/163`.

[35] Alfonso Torres-Ruiz and Robert W. Jones. Comparison of the efficiency of the bumble bees *Bombus impatiens and Bombus ephippiatus* (Hymenoptera: Apidae) as pollinators of tomato in greenhouses. *Journal of Economic Entomology*, 105(6):1871–1877, December 2012. ISSN 0022-0493. doi: 10.1603/EC12171. URL `https://doi.org/10.1603/EC12171`.

[36] James P Strange, Amber D Tripodi, Thuy-Tien T Lindsay, James D Herndon, Joyce Knoblett, Morgan E Christman, N Pinar Barkan, and Jonathan B U Koch. Variation in North American bumble bee nest success and colony sizes under captive rearing conditions. *Journal of Insect Science*, 23(3):10, May 2023. ISSN 1536-2442. doi: 10.1093/jisesa/iead032. URL `https://doi.org/10.1093/jisesa/iead032`.

[37] Kathryn E. Gardner, Robin L. Foster, and Sean O'Donnell. Experimental analysis of worker division of labor in bumblebee nest thermoregulation (*Bombus huntii*, Hymenoptera: Apidae). *Behavioral Ecology and Sociobiology*, 61(5):783–792, March 2007. ISSN 1432-0762. doi: 10.1007/s00265-006-0309-7. URL `https://doi.org/10.1007/s00265-006-0309-7`.

[38] James H. Cane. The extraordinary Alkali Bee, *Nomia melanderi* (Halictidae), the world's only intensively managed ground-nesting bee. *Annual Review of Entomology*, 69(Volume 69, 2024):99–116, January 2024. ISSN 0066-4170, 1545-4487. doi: 10.1146/annurev-ento-020623-013716. URL `https://www.annualreviews.org/content/journals/10.1146/annurev-ento-020623-013716`.

[39] Ryan J. Leonard and Alexandra N. Harmon-Threatt. Methods for rearing ground-nesting bees under laboratory conditions. *Apidologie*, 50(5):689–703, October 2019. ISSN 1297-9678. doi: 10.1007/s13592-019-00679-8. URL `https://doi.org/10.1007/s13592-019-00679-8`.

[40] Jordi Bosch and William P Kemp. The life cycle of *Osmia lignaria*: implications for rearing populations. *Solitary bees. Conservation, rearing and management for pollination. Imprensa Universitária, Fortaleza*, pages 153–160, 2004.

[41] Aleksandra Splitt, Schulz , Michał, , and Piotr Skórka. Current state of knowledge on the biology and breeding of the solitary bee – *Osmia bicornis*. *Journal of Apicultural Research*, 61(2):163–179, March 2022. ISSN 0021-8839. doi: 10.1080/00218839.2021.1957610. URL https://doi.org/10.1080/00218839.2021.1957610. _eprint: https://doi.org/10.1080/00218839.2021.1957610.

[42] Kyeong Yong Lee, Hyung Joo Yoon, Kwang Sik Lee, and Byung Rae Jin. Development and mating behavior of *Osmia cornifrons* (Hymenoptera: Megachilidae) in the constant temperature. *Journal of Asia-Pacific Entomology*, 19(2):281–287, June 2016. ISSN 1226-8615. doi: 10.1016/j.aspen.2016.03.003. URL https://www.sciencedirect.com/science/article/pii/S1226861516300346.

[43] Petr Bogusch, Lucie Hlaváčková, Karel Šilhán, and Michal Horsák. Long-term changes of steppe-associated wild bees differ between shell-nesting and ground-nesting species. *Journal of Insect Conservation*, 24(3):513–523, March 2020. ISSN 1366-638X. doi: 10.1007/s10841-020-00232-4. URL http://dx.doi.org/10.1007/s10841-020-00232-4.

[44] R. W. Rust. Biology of *Osmia (Osmia) ribifloris* Cockerell (Hymenoptera: Megachilidae). *Journal of the Kansas Entomological Society*, 59(1):89–94, 1986. ISSN 0022-8567. URL https://www.jstor.org/stable/25084741.

[45] Vincent J Tepedino and Frank D Parker. Effect of rearing temperature on mortality, second-generation emergence, and size of adult in *Megachile rotundata* (Hymenoptera: Megachilidae). *Journal of Economic Entomology*, 79(4):974–977, 1986.

[46] Salvatore Vicidomini. Biology of *Xylocopa (Xylocopa) violacea* (L., 1758)(Hymenoptera: Apidae): nest morphology. *Atti Soc. Ital. Sci. Nat. Milano*, 136(2):95–108, 1995.

[47] Adna Suelen Dorigo, Annelise de Souza Rosa-Fontana, Hellen Maria Soares-Lima, Juliana Stephanie Galaschi-Teixeira, Roberta Cornélio Ferreira Nocelli, and Osmar Malaspina. In vitro larval rearing protocol for the stingless bee species *Melipona scutellaris* for toxicological studies. *PLOS ONE*, 14(3):e0213109, March 2019. ISSN 1932-6203. doi: 10.1371/journal.pone.0213109. URL https://journals.plos.org/plosone/article?id=10.1371/journal.pone.0213109.

[48] José de Ribamar Silva Barros. Genetic breeding on the bee *Melipona scutellaris* (Apidae, Meliponinae). *Acta Amazonica*, 36:115–120, March 2006. ISSN 0044-5967, 1809-4392. doi: https://doi.org/10.1590/S0044-59672006000100014. URL https://www.scielo.br/j/aa/a/GPZWQgvhvXNDNdzz8pnbDKg/?lang=en.

[49] Kamila Leão Leão, Ana Carolina Martins de Queiroz, Jamille Costa Veiga, Felipe Andrés León Contrera, and Giorgio Cristino Venturieri. Colony development and management of the stingless bee *Scaptotrigona aff. postica* (Apidae, Meliponini) in different hive models. *Sociobiology*, 63(4):1038–1045, 2016.

[50] D Koedam, v Velthausz, PH, T vd Krift, MR Dohmen, and MJ Sommeijer. Morphology of reproductive and trophic eggs and their controlled release by workers in *Trigona (Tetragonisca) angustula* llliger (Apidae, Meliponinae). *Physiological Entomology*, 21(4):289–296, 1996.
